# TrakEM2 Software for Neural Circuit Reconstruction

**DOI:** 10.1371/journal.pone.0038011

**Published:** 2012-06-19

**Authors:** Albert Cardona, Stephan Saalfeld, Johannes Schindelin, Ignacio Arganda-Carreras, Stephan Preibisch, Mark Longair, Pavel Tomancak, Volker Hartenstein, Rodney J. Douglas

**Affiliations:** 1 Institute of Neuroinformatics, University of Zurich and ETH Zurich, Zurich, Switzerland; 2 Max Planck Institute of Molecular Cell Biology and Genetics, Dresden, Germany; 3 Massachusetts Institute of Technology, Boston, Massachusetts, United States of America; 4 Molecular Cell and Developmental Biology Department, University of California Los Angeles, Los Angeles, California, United States of America; Harvard University, United States of America

## Abstract

A key challenge in neuroscience is the expeditious reconstruction of neuronal circuits. For model systems such as *Drosophila* and *C. elegans*, the limiting step is no longer the acquisition of imagery but the extraction of the circuit from images. For this purpose, we designed a software application, TrakEM2, that addresses the systematic reconstruction of neuronal circuits from large electron microscopical and optical image volumes. We address the challenges of image volume composition from individual, deformed images; of the reconstruction of neuronal arbors and annotation of synapses with fast manual and semi-automatic methods; and the management of large collections of both images and annotations. The output is a neural circuit of 3d arbors and synapses, encoded in NeuroML and other formats, ready for analysis.

## Introduction

There is a growing consensus that detailed volumetric reconstructions of thousands of neurons in millimeter-scale blocks of tissue are necessary for understanding neuronal circuits [1], [2]. Modern electron microscopes (EM) with automatic image acquisition are able to deliver very large collections of image tiles [3]–[8]. Unfortunately, the problems of acquiring the data have so far been easier to solve than that of interpreting it [9], . Increasingly, neuroscience laboratories require automated tools for managing these vast EM data sets using affordable consumer desktop computers.

Here, we present such a tool. It is an open source software package, named TrakEM2, that is optimised for neural circuit reconstruction from tera-scale serial section EM image data sets. The software handles all the required steps: rapid entry, organization, and navigation through tera-scale EM image collections. Semi- and automatic image registration is easily perfomed within and across sections. Efficient tools enable manipulating, visualizing, reconstructing, annotating, and measuring neuronal components embedded in the data. An ontology-controlled tree structure is used to assemble hierarchical groupings of reconstructed components in terms of biologically meaningful entities such as neurons, synapses, tracts and tissues. TrakEM2 allows millions of reconstructed entities to be manipulated in nested groups that encapsulate the desired abstract level of analysis, such as “neuron”, “compartment” or “neuronal lineage”. The end products are 3D morphological reconstructions, measurements, and neural circuits specified in *NeuroML* [11] and other formats for functional analysis elsewhere.

TrakEM2 has been used successfully for the reconstruction of targeted EM microvolumes of *Drosophila* larval central nervous system [7], for array tomography [12], for the reconstruction and automatic recognition of neural lineages in LSM stacks [13], for the reconstruction of thalamo-cortical connections in the cat visual cortex [14] and for the reconstruction of the inhibitory network relating selective-orientation interneurons in a 10 Terabyte EM image data set of the mouse visual cortex [8], amongst others.

## Results

### From Raw Collections of 2d Images to Browsable Recomposed Sample Volumes

**Figure 1:**
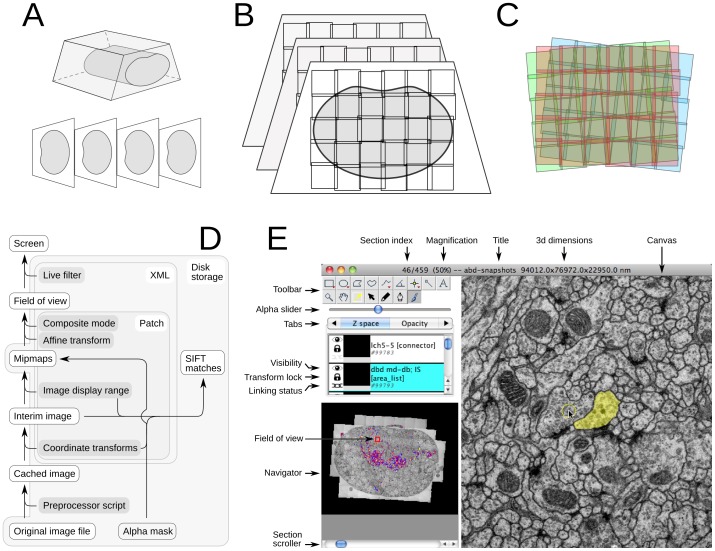
From a resin block to serial 2d image montages. **A** Serial EM is performed on a block of tissue embedded in hardened plastic resin. **B** Sections are imaged with multiple overlapping image tiles. **C** The imprecision in the positioning of the camera and the numerous non-linear deformations demand of an automatic multi-section image registration procedure that computes the best possible transformation for each tile without introducing gross deformations. **D** TrakEM2 operates only on original images, which are treated as read-only. A *preprocessor script* specified invidually for every image alters the image after loading from disk and before the rest of TrakEM2 has access to it, enabling changes of scale, of look-up table, data type, and any pixel-level operation. A *Patch* object encapsulates the image file path and a set of properties such as the alpha mask, the coordinate transforms (linear and non-linear image transformations) and the desired image display range and composite mode, among others. The precomputed mipmaps store most of the *Patch* information in compressed 8-bit files ready for display. The image for the field of view is constructed from composing multiple *Patch* instances according to their location and composite rules (overlay, subtract, add, multiply, difference and Colorize YCbCr), and is then filtered, if desired, for dynamic interactive image enhancement. **E** The TrakEM2 *Display* presents the field of view showing a single section and the images, segmentations and annotations present in that section. The *Display* provides access to tools for manipulating and analyzing all imported images and reconstructed elements.

An EM volume large enough to encapsulate significant fractions of neuronal tissue and with a resolution high enough to discern synapses presents numerous challenges for visualization, processing and annotation. The data generally consists of collections of 2d image tiles acquired from serial tissue sections (Figure 1; [7], [8]) or from the trimmed block face (Block-face Serial EM or SBEM, [3], [15]; focused ion beam scanning EM or FIBSEM, [6]) that are collectively far larger than Random Access Memory (RAM) of common lab computers and must be loaded and unloaded on demand from file storage systems. Additional experiments on the same data sample may have generated light-microscopical image volumes that must then be overlaid on the EM images, such as in array tomography [12], [16] or correlative calcium imaging [8], [15]. TrakEM2 makes browsing and annotating mixed, overlaid types of images (Figure S1) over terabyte-sized volumes fast (Text S1, section “Browsing large serial EM image sets”) while enabling the independent manipulation of every single image both from a point-and-click graphical user interface (GUI; Figure 1e, S2, S3, S4) and by automatic means (Text S1, section “Image adjustment”).

The images acquired with the EM microscope represent views of tissue that has been deformed by the sectioning process, by the heat of the electron beam, by charging effects, and by the magnetic lenses. For serial sections, part of the section may be hidden away by a section fold or support-film fold (Figure S5), and counterstaining with heavy metals further increases the difficulty of the task by occluding parts of the section with accidental precipitates (Figure S5). All images require illumination adjustments (Figure S5, S6).

TrakEM2 recovers the original sample present in the resin block from the images with a robust automatic multi-step image registration approach. First images are corrected for distortions induced by the EM magnetic lenses [17]. Then, image tiles belonging to individual sections are montaged combining a linear alignment established from invariant image features (SIFT; [18]) and an elastic alignment that compensates for the remaining non-linear distortion [19].

Similarly, the section series are aligned by firstly using invariant features to estimate a linear transformation followed by elastic alignment to compensate for non-linear distortion. Alternatively to an immediate elastic alignment of the series of montages, feature correspondences can be used to estimate each image tile’s globally optimal pose with respect to overlapping tiles within the same section and in adjacent sections [20]. This method enables the reconstruction of section series from section montages that cover only a few regions of interest disconnected in the section plane but related across sections (e.g. sparse images of different branches of a neuron). The methods implemented for montaging, global tile pose estimation and elastic alignment calculate global alignments for groups of images while explicitly minimizing the local deformation applied to each single image. Only by that constraint, very large montages or series of montages can be aligned without accumulating artificial deformation [19].

In combination, TrakEM2’s alignment and deformation correction tools both manual and automatic allow high quality volume reconstruction from very large section series. Complex imaging arrangements are supported, including low-resolution images of large fields of view that were then complemented with high-resolution images for areas of interest, or different tilts of the same section. Tens of thousands of images are registered with an off-the-shelf computer in a few days.

Both linear and non-linear transformations are expressed with a system that brings pixels from the original image space to the transformed space in one single computational step, concatenating all transformations and expressing the final transformation in the precomputed mipmap images (Figure 1d; Text S1, section “Browsing large serial EM image sets”). Additionally, the TrakEM2 GUI enables direct point-and-click manipulation of the transformation of any image in the volume, before or after the automatic registration without significant cost in data storage (relative to the dimensions of the image) or image quality (Text S1, section “Assembling the volume with automatic registration of image tiles” and “Manually correcting automatic image registration with affine and non-linear transformations”; Figure S2, S3).

### Reconstructing a Neuronal Circuit from an Image Volume

The second step in neuronal circuit reconstruction consists in identifying and labeling the neurons and synapses in the image volume. The current gold standard is computer-assisted manual labeling, either by brushing 2d areas ([7], [21]; not practical for large volumes) or by marking skeletons [8], [15], [22]. Automated methods for neuronal reconstruction are currently the focus of intensive research in Computer Vision (for review see [9]). TrakEM2 offers manual and semi-automatic methods for image segmentation (Figure S7) and for sketching structures with *spheres* and *tubes* (Text S1, section “Stick-and-ball models”; Figure S8), and interfaces with automatic image segmentation programs (Text S1, section “Image segmentation for 3d object reconstruction”).

**Figure 2:**
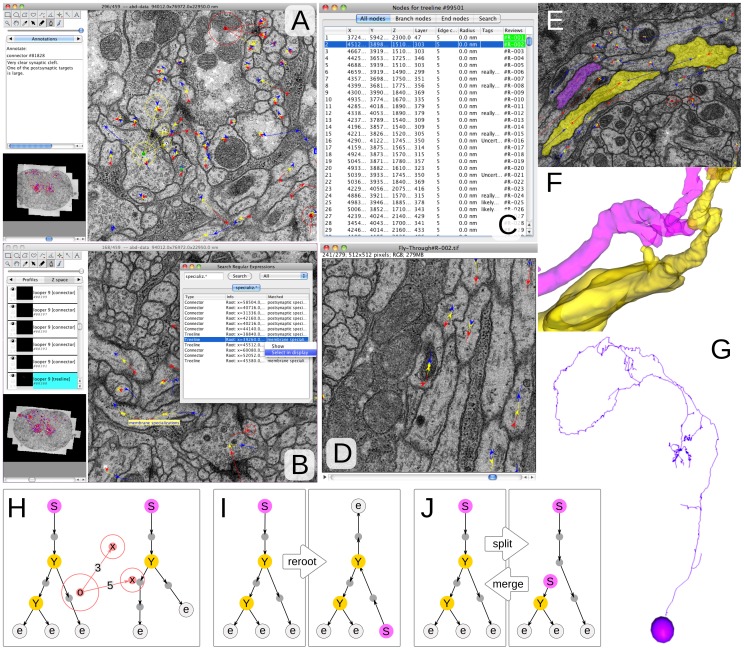
Neural circuit reconstruction with skeletonized neural arbors and connectors to relate them at synaptic sites. **A** Snapshot illustrating the use of connectors to relate neural arbors. The connector in green (notice the ‘o’ node with a yellow circle around; it has three targets–it’s a polyadic insect synapse), each of which is represented within the section by a node with an arrow head that falls within the circle of each target. To the left, notice the use of text annotations to describe the synapse. **B** Search with regular expressions locates any objects of interest, in this case a “membrane specializations” tag in a neuronal arbor. **C** The tabular view for a neural arbor lists all nodes, branch nodes, end nodes or a subset whose tags match a regular expression. All columns are sortable, and clicking on each row positions the display on the node. The last column titled “Reviews” indicates which cables of the neuron have already been reviewed (in green) to correct for missing branches or synapses or other issues. **D** A review stack is precomputed for fast visualization of the cable of interest, each section centered on the node. The visual flow through the stack helps in catching reconstruction errors. **E** “Area trees” are skeleton arbors whose nodes have 2d areas associated. **F** 3d rendering of two “area trees”, a section of which are depicted in E. **G** 3d rendering of the nucleus (represented by a “ball”) and the arbor (represented by a “treeline”) of a neuron in the insect brain. **H–J** Cartons of the skeletons used for reconstruction. The root node is labeled with an “S”, the branch nodes with “Y” and the end nodes with “e”. In H, a “connector” relates the nodes of two arbors, with specific confidence value for the relationship. These confidence values exist on the edges that relate the arbor’s nodes as well (not shown). **I** Rerooting changes the perspective, but not the topology, of the tree. By convention we position the root node at the soma. **J** Two common and trivial operations on trees are split and merge.

Manual skeletonization of a neuronal arbor requires continuous recognition operations that are not always done with full confidence given ambiguity in the image data. In our experience an all-or-nothing approach (edge or no edge, that is, to connect two parts of a neuronal arbor or not) does not sufficiently express all the information available to the human operator. Therefore TrakEM2’s *skeleton* data types are composed of nodes and directional edges that express parent/child relationships between nodes with a confidence value that captures the degree of certainty in the continuity of the skeleton at that edge (Figure 2). Edge confidence values are particularly useful to restrict ulterior circuit analysis to the most trustable subsets of the skeletons. Additionally each node holds a list of text annotations (“tags”) to highlight structures of interest or to label nodes as places to branch out later (e.g. with a *TODO* tag), and also a radius value (*treeline* skeleton subtype) or a 2d area (*areatree* skeleton subtype) to render 3d skeletons as stick models or volumes, respectively (Figure 2; Text S1, section “Image segmentation for 3d object reconstruction”). To correct mistakes skeletons are cut or joined at any node. Node edges accept any color (e.g. to label a branch), or follow a color code that expresses betweeness-centrality (computed as in [23]) relative to other nodes, branches or synapses.

Given the unreliability of human-based skeletonization (*tracing*) of neurons [22], TrakEM2 facilitates the revision of skeleton nodes. An interactive GUI table lists all skeleton nodes and sorts them by location, edge confidence or tags, allowing quick targeted review of interesting or problematic parts of the skeleton (Figure 2). To systematically review complete neuronal arbors, TrakEM2 generates sequences of images centered at each node (*fly-throughs*) for each skeleton branch (Figure 2) that exploit the human ability to detect small changes in optic flow: missassignments across sections are readily identified as sudden shifts in the field of view. This review method aids as well in locating unlabeled synapses and untraced branches.

TrakEM2 expresses synapses with *connector* elements that relate areas or skeleton nodes with other areas or nodes. Each *connector* consists of an origin and a number of targets, each assigned a confidence value, to express from monadic to diadic and polyadic synapses (Figure 2h). To aid the systematic reconstruction of all upstream and downstream neuron partners of a specific neuron, TrakEM2 presents an interactive table that lists all the incoming and outgoing connectors of a skeleton, and who they connect to. Incomplete synaptic partners are then visited one at a time and reconstructed. All tables are dynamically updated as nodes and connectors are added to or removed from the skeletons. The resulting neuronal circuit is then exported in various formats including NeuroML [11].

### Structuring Reconstructions Hierarchically with Semantically Meaningful Groups

The reconstruction of one or a few neuronal arbors is very different to the reconstruction of a complete neuronal processing module. The main difference is the scale: the latter is generally composed of dozens or thousands of neuronal arbors. While a human operator tracks the identities of a small collection of elements with ease, the task becomes very time consuming and error prone for large collections of neurons. In our experience the cut off is at about 50 elements.

**Figure 3:**
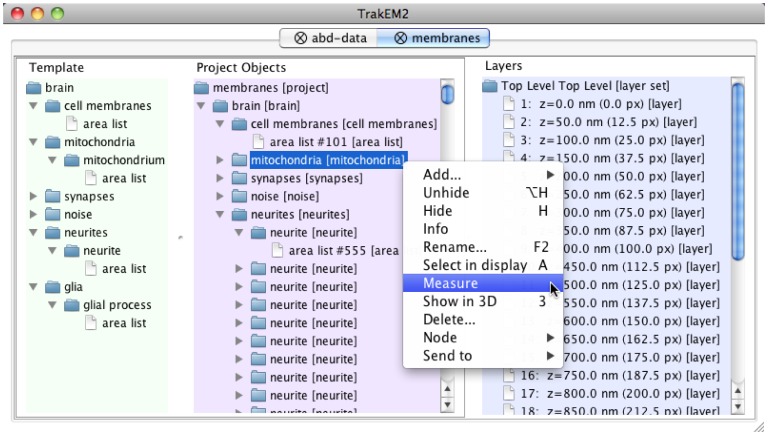
Hierarchical organization of reconstructed objects. A *Template* (*to the left*) restricts the expression of nested abstract concepts (such as “brain”, “mitochondria”, etc.) and indicates what other abstract types (e.g. a “glia” is represented by one or more “glial process” instances) or primitive types (such as “area list”, “treeline”, “connector”, “ball”, etc) they may be represented with. All elements of the *Template* are specific of each reconstruction project and user-defined. In the center, *Project Objects* displays the actual instances of the abstract, templated objects, which encapsulate and organize in many levels of abstract types the primitive segmentation types (e.g. “area list”). The hierarchical structure assigns meaning to what otherwise would be an unordered heap of primitive types. Each instance of a primitive type acquires a unique identifier (such as “#101 [area list]” ). Each group may be measured jointly, or visualized in 3d, shown/hidden, removed, etc., as illustrated in the contextual menu for the selected “mitochondria” group (*highlighted in blue*). To the right, the *Layers* list all sections in the project (a “Layer” holds the data for a single tissue section). From this graphical interface, an independent view may be opened for each section.

Nesting arbitrary groupings of reconstructed elements collapses a collection of arbitrary reconstructions into a meaningful entity such as a neuron. For example, a neuron may be represented with a nucleus (represented by a *sphere*), an arbor (represented by an *areatree*) and a list of synapses (each represented by a *connector*). Large collections of neurons are grouped by modality (“sensory neurons” versus “motor neurons” or “interneurons”), or by lineage (such as “BLD5”, “DALcl2”, etc. in the fly larval brain), or by experimental condition (“GFP-labeled”, “RFP-labeled”), or by any desirable arbitrary grouping or nested groupings. Hierarchical grouping effectively reduces the complexity in the management of large collections of objects by collapsing them into high-level entities meaningful for the human researcher. These groups are application-specific and in TrakEM2 are constrained by a controlled vocabulary with the required hierarchical groups (Figure 3). With hierarchical data organization and a search tool that supports regular-expressions, TrakEM2 enables the location, manipulation, measurement (Text S1 “Measurements”; Figure S9) and visualization of entities at the desired level of abstraction, be it fragments of neurons, individual neurons, a lineage of neurons, neuronal circuits, or arbitrary compartments or areas of the brain.

## Discussion

We have described the key properties of TrakEM2, an open source software that is optimized for neural circuit reconstruction from serial section EM image data sets. TrakEM2 answers the quickly growing demand for a flexible and robust application for implementing at tera-scale the workflows typical of current connectomics projects that require volumetric reconstruction, visualization, and analysis of objects observed through 2D images. In this way, TrakEM2 supports the quest of neuroscientists to obtain a complete picture of the circuits embedded in the densely connected neurons of nervous systems. Indeed, ever since Schwann’s theory of the cell and Cajal’s neuron doctrine, neuroscientists have struggled to describe the diversity of neurons in the brain and their synaptic contacts that define the neuronal circuitry underlying brain functions.

The turning point in this quest occurred in 1986, when Sydney Brenner and collaborators published their monumental work, the complete wiring diagram of the nematode *Caenorhabditis elegans*, with only 302 neurons [24]. The choice of organism was key to their success, given the technological means of the time. However, a quarter of a century later, no other central nervous system has been reconstructed in full.

Brenner’s reconstruction of the *C. elegans* nervous system was performed largely without the assistance of a computer. The work consisted in photographing (with film) serial 50 nanometer sections of the nematode worm, and annotating neurons and synapses on paper prints. An early computer-based system [25] was used for three-dimensional reconstruction of a few very small volumes. The introduction of personal computers in the mid-eighties opened the way for the development of the first computer-assisted reconstruction systems such as TRAKA [26] and three years later Neurolucida ([27]; MicroBrightField), bringing feasibility to computer-assisted neuronal reconstruction. Both these systems were oriented towards the reconstruction of labeled neurons at the optical level. They solved the data storage problem of the time, that very large fields of view were far too large for computerized storage, by operating on microscope stage coordinates rather than pixel coordinates in a digitized image. Meanwhile, the results of Moore’s Law, and improving electronic camera technology, have opened opportunities for storing and manipulating very large datasets of images. For large-scale serial section electron microscopy (EM) in its many variants (serial section electron tomography or SSET, [28]; serial section transmission EM or ssTEM; block-face EM or SBEM [3]; focused ion beam scanning EM or FIBSEM, [6]), coupling live imaging with neuronal reconstruction would result in damage to, and eventually disruption of, the nanometer-thick sections, or it is not possible (such as in block-face EM or FIBSEM). Acquiring images first and then performing the analysis offline is necessary.

The software IMOD [29] revolutionized EM image volume analysis with tools for visualizing and aligning the sections of image stacks, and for manually counting, measuring and modeling objects in the 3d volume. The software Reconstruct [21] catered to the special needs of neuronal reconstruction from EM, namely tools for manual and semi-automated image registration within a section (montaging, for large fields of view) and across serial sections, and tools for volumetric reconstruction and measurement of neuronal structures. The software package ir-tools [30] made new developments of the computer vision field accessible for serial EM reconstructions, including automated image montaging and contrast limited adaptive histogram equalization for image enhancement (CLAHE; [31]), among others. All these softwares evolved considerably since their publication dates and complement each other to various degrees. Originally, each was designed with specific technological problems and scientific questions in mind.

TrakEM2 is deployed along with all the necessary image processing libraries with Fiji [32], an open source image processing application. Fiji provides automatic deployment of software updates and comprehensive documentation via a publicly accessible wiki (http://pacific.mpi-cbg.de). Fiji supports a variety of scripting languages useful for the programmatic manipulation of data structures in TrakEM2. The functionality and batch-processing capabilities of TrakEM2 are extensible at will.

TrakEM2 has already been employed in a variety of applications. While originally designed for reconstructing neural circuits in anisotropic serial section EM (for example, see [7], [8], [14]), researchers have found TrakEM2 useful for other EM modalities, for example for registering series of images from FIBSEM and annotating synapses by hand [33]. The segmentation tools have been used for generating a gold standard segmentation of brain tissue to compare with the output of automatic segmentation algorithms on EM images [34], and for reconstructing neuronal lineages [7] and organs [35] in laser-scanning microscopy data sets.

TrakEM2 must evolve as new imaging methods deliver higher-resolution data sets of ever increasing volumes. The open source nature of TrakEM2 allows any researcher to modify the program to suit specialized needs, and to incorporate implementations for novel algorithms from the computer vision and image processing fields. For example, TrakEM2 currenty exploits the anisotropic nature of serial section EM data, in which the X and Y dimensions have about 10 times higher resolution than Z (which is limited by the thickness of the section). Now, novel algorithms for tomographic reconstruction of serial sections [36] and more isotropic EM imaging with BFSSEM [3] and FIBSEM [6] suggest that the approach, which limits the manipulation of image data to the XY plane will need to evolve to meet this challenge. General improvements in data storage and computing capacity will be very helpful for handling the coming new kind of large isotropic high-resolution EM data sets.

TrakEM2 source code is under a distributed version control system (git) that encourages forking the source code base, while retaining the capability of contributing back to the main development branch. TrakEM2 has been publicly available as open source since day one. The many contributions of interested users and developers have, and will, greatly enhance the utility of TrakEM2, for the benefit of all.

## Materials and Methods

### Source Code

TrakEM2 has been written using the Java programming language and uses numerous image processing libraries including ImageJ (Wayne Rasband), mpicbg (Stephan Saalfeld), LOCI bio-formats [37], ImgLib (Stephan Preibisch, Stephan Saalfeld, Tobias Pietzsch and others), ImageJ 3D Viewer [38], Stitching [39], bUnwarpJ [40], JaMa (Mathworks and NIST), postgresql-jdbc, JFreeChart (jfree.org), edu_mines_jtk (Dave Hale), Level Sets (Erwin Frise) and Simple Neurite Tracer [41], among others. The source code is released under the General Public License and is under version control with git at http://repo.or.cz/w/TrakEM2.git. Binaries are distributed with Fiji (Schindelin et al, submitted to Nature Methods) via the automatic plugin updater.

### Example EM Data

The EM data used here to exemplify the use of TrakEM2 corresponds to the abdominal neuropil of the first instar larva of *Drosophila*, and will be made available in full elsewhere.

## Supporting Information

Figure S1 **Section and image compositing rules for simultaneous visualization of multiple sections or multiple channels.** **A** Three consecutive sections (called *Layer* in TrakEM2 parlance), each with numerous tiles, are simultaneously rendered in red (previous), green (current) and blue (next). The gray area indicates that the overlap is very good. **B** The previous section is overlaid using a ‘difference’ composite: regions of the image that do not match will get highlighted in white. **C** RGB image tile from an antibody labeling manually registered on top of a collection of montaged EM tiles using a Color YCbCr composite. **D** Higher magnification of a similar region shown in C, where specific sectioned axons and dendrites are seen labeled in red or green. The overlay greatly facilitates identifying neurons in reasonably stereotypical animals such as *Drosophila*.
(PDF)

Figure S2 **Manual affine transform of collections of image tiles.** **A** The affine transform mode is used for interactive multi-tile transformations. In conjunction with multi-section visualization (the editable section in green, and the previous, reference section in red–the best overlap in yellow), a section is manually aligned to the previous–a capability most useful for correcting or refining the results of automatic registration algorithms. *A2* Enlarged inset, revealing the lack of overlap of the two adjacent sections. Notice near top right how the green section doesn’t overlap with the red section. Three landmarks that define an affine transformation are used to interactively adjust the pose of all tiles in the section. **B, B2** After manually dragging the landmark the two sections now overlap more accurately. The transformation is then propagated to subsequent sections to preserve the relative pose of all tiles (see menu snapshot in **A**).
(PDF)

Figure S3 **Manual non-linear transform of collections of image tiles for fine cross-section alignment.** **A,B** Two consecutive sections numbered 344 and 345 present an artefactual stretch, as indicated by the widening of the marked profiles (in white). **C,D** The manual non-linear transformation mode is used here in conjunction with the transparent section overlay (notice the slider above the green panel in **C**) to reveal the local misalignment. The inset in **C,D** indicates the local transformation performed by dragging numerous landmarks.
(PDF)

Figure S4 **Expressing image transformations without duplicating the original images by using alpha masks.** Duplicating images has a huge cost in data storage which TrakEM2 avoids by using highly compressible alpha masks and precomputed mipmaps stored with lossy compression. **A** Images present borders which are apparent when overlapping (red arrowheads). An alpha mask with zero values for the borders (see adjacent cartoon) removes the border from the field of view. A1 and A2 images show the rectangular region marked in red in the cartoons. **B** Manual non-linear transformations before (A1) and after (A2) corrects a section fold in an image tile. *Inset*, the alpha mask of the corrected tile. **C** Alternatively, the manual image splitting mode cuts image tiles in two or more parts using a polygonal line (C1), so that each half is now an independent *Patch* object that represents a tile, each relying on the original image but with a different alpha mask (inset in C2). Rigid image registration may now proceed, visualized in C3 by overlaying two consecutive sections. Data in B and C courtesy of Ian Meinertzhagen, Dalhousie University (Canada).
(PDF)

Figure S5 **Correctable noise on EM images.** **A1, A2** A large blob occludes information on an EM image when the display range is adjusted for the whole image (A1), but reveals its content when CLAHE is applied (A2). **B1-4** A support-film fold generates a dark band (B1) whose content is discernible at a lower value region of the histogram (inset in B2). Applying CLAHE with a small window partially solves the problem (B3) but composing the image from both ranges restores it best (B4).
(PDF)

Figure S6 **On-the-fly processing of the field of view for enhanced contrast.** The live filter tab of the display offers a few filters, to adjust **A** the display range; invert the image (not shown) or **B** CLAHE. Yellow rectangle indicates the original view without filters.
(PDF)

Figure S7 **Volumetric reconstruction with series of complex 2d areas or “area lists”.** The “Z space” tab lists all segmentation objects that exist in 3d. **A** With the *brush* tool, a selected “area list” instance is painted in yellow (notice the mouse pointer with circle), labeling the sectioned profile of a neuron. The selected object (listed in the cyan panel) may be visible or hidden, locked, or linked to the underlying images. **B** Labeled meshes are rendered in 3d by generating a mesh of triangles with marching cubes. **C** Dense reconstruction of a cube of neuropil.
(PDF)

Figure S8 **Sketching and quantifying neural tissue with spheres and tubes.** **A,B** Two sections with a “ball” to represent the nucleus and a “pipe” to model the main process of a monopolar insect neuron. The colors indicate relative depth: red means below the current section and blue above. **C** 3d representation of the “ball” and “pipe” traversing multiple sections. **D** Usage of “ball” sketching type for quantifying the number of synaptic vesicles. The synaptic cleft is modeled with an “area list”. **E** 3d representation of the synaptic vesicles and cleft modeled in D. **F** Results table with the count and position of labeled vesicles. Data in D,E courtesy of Graham Knott, EPFL (Switzerland).
(PDF)

Figure S9 **Measurements.** **A** Example of a “connector” instance, expressing a synapse between an axon (large profile at lower left with numerous microtubules) whose tree is tagged “presynaptic site”, with numerous terminal dendrites (small target circles, one in red indicating it’s in the previous section). **B** Measurement of the distances from the root node (the soma, by convention) to all nodes labeled “presynaptic site” like in A. The inset schematizes the measurements (dotted red lines from “root” to “nodes labeled as “pre”). **C** A double disector is used together with an overlay grid (in green, cell size is one micron) to detect the number of objects appearing new in the next section (objects labeled as little yellow squares, with blue circles for the position of the same object in the next section, if present). The table shows the list of all marked objects. Note how “3″ occurs only once, indicating that it appears new in the next section. See [42] for details on the double disector technique. **D** The built-in scripting editor in Fiji shows a small python script to extract statistics on the distances of synaptic vesicles (modeled with a “ball”) to a synaptic cleft (modeled with an “area list”), as shown in Supplemental Figure 11 d, e.
(PDF)

Text S1 **Supplemental Text containing detailed information on various aspects of the TrakEM2 software, including image registration, dealing with noise, alpha masks, manual segmentation with areas, balls and pipe objects, and measurements.**
(PDF)
