# Supplementary figures and images for: TrakEM2 Software for Neural Circuit Reconstruction
Source: PLoS One. 2012 Jun 19;7(6):e38011. doi: 10.1371/journal.pone.0038011 (PMC3378562; doi:10.1371/journal.pone.0038011)

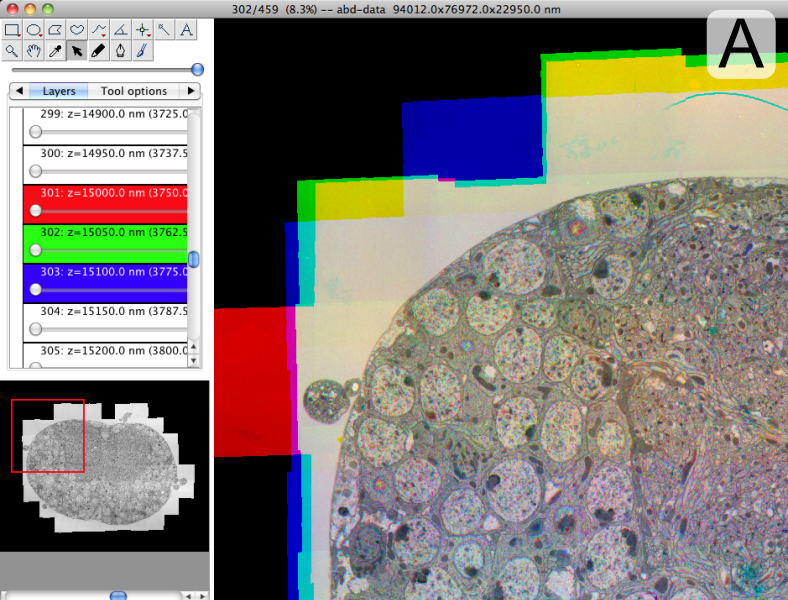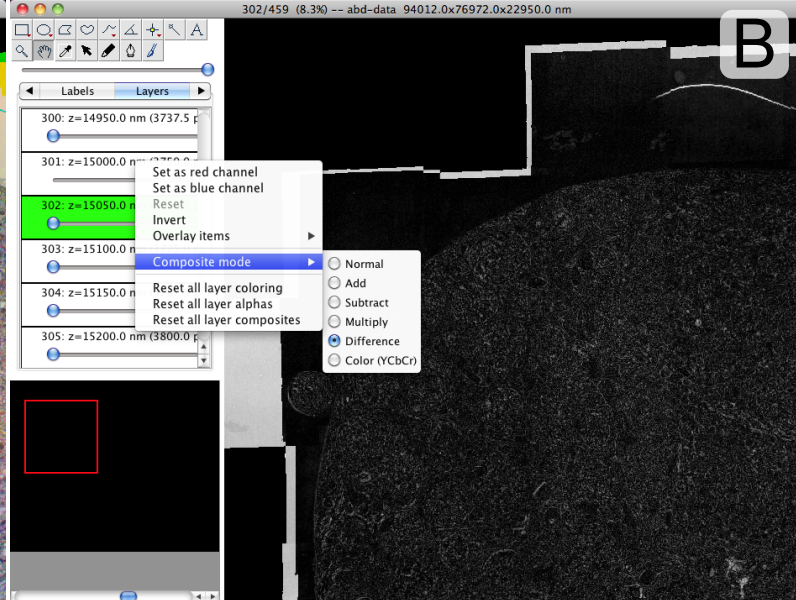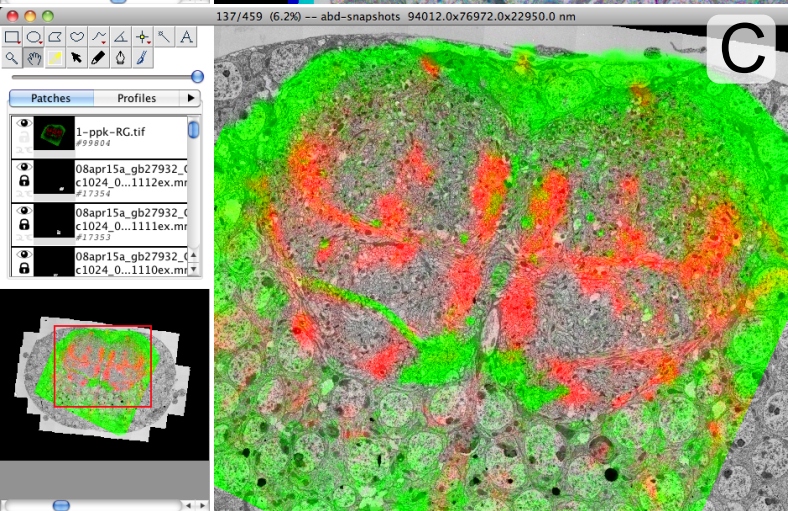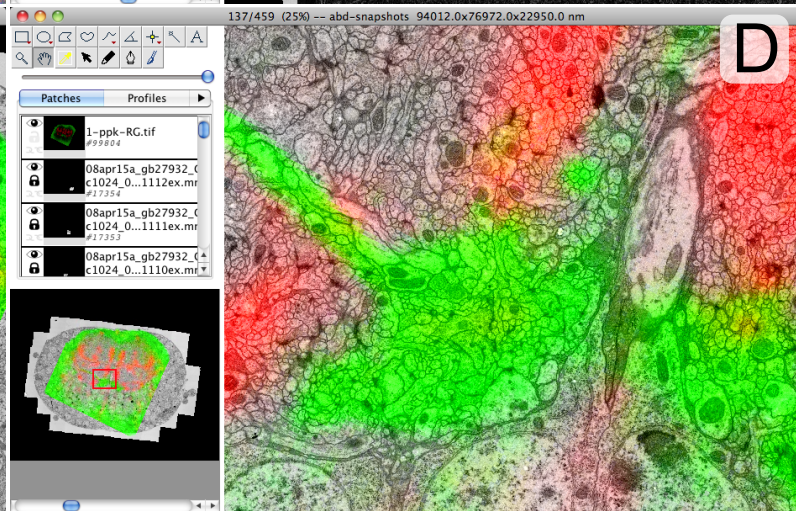

Supplement: Figure S1 — Section and image compositing rules for simultaneous visualization of multiple sections or multiple channels. A Three consecutive sections (called Layer in TrakEM2 parlance), each with numerous tiles, are simultaneously rendered in red (previous), green (current) and blue (next). The gray area indicates that the overlap is very good. B The previous section is overlaid using a ‘difference’ composite: regions of the image that do not match will get highlighted in white. C RGB image tile from an antibody labeling manually registered on top of a collection of montaged EM tiles using a Color YCbCr composite. D Higher magnification of a similar region shown in C, where specific sectioned axons and dendrites are seen labeled in red or green. The overlay greatly facilitates identifying neurons in reasonably stereotypical animals such as Drosophila. (PDF) [file pone.0038011.s001.pdf]

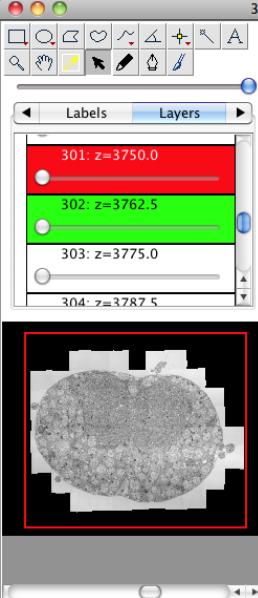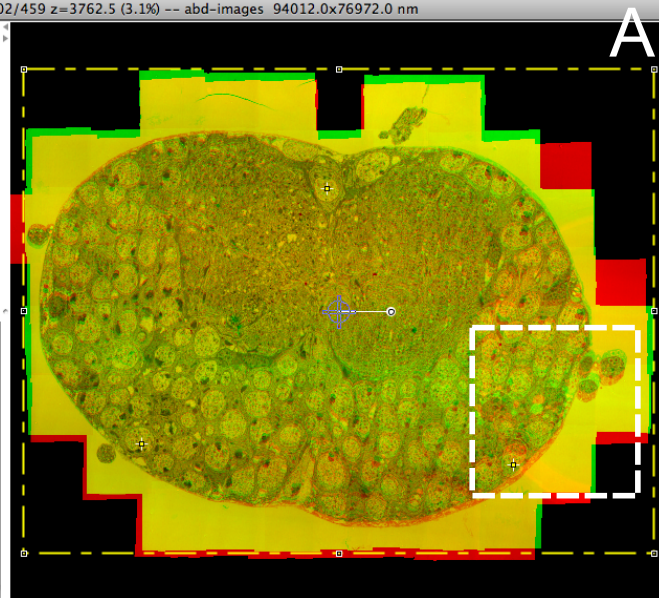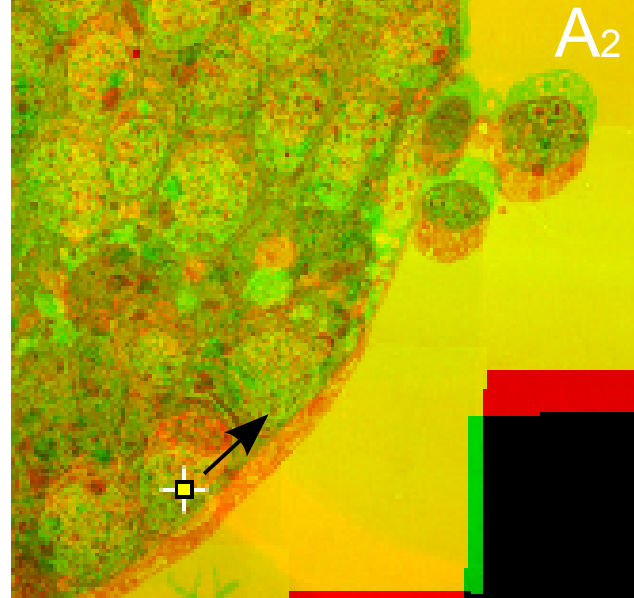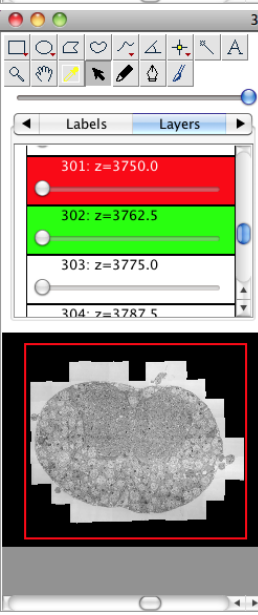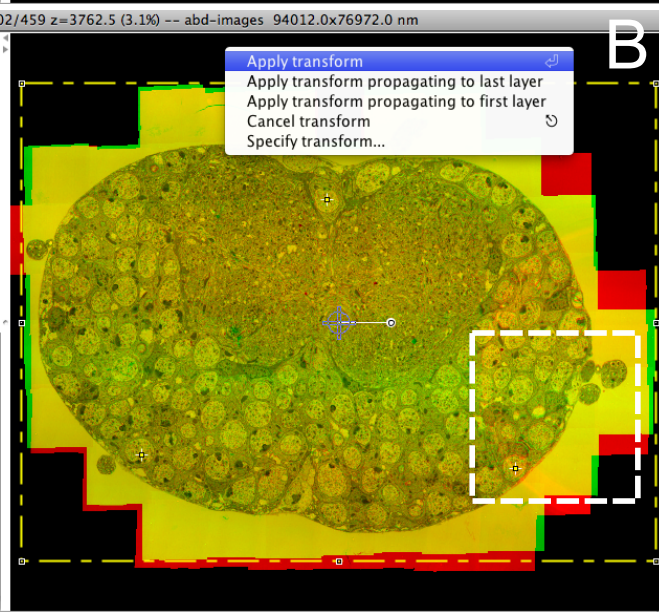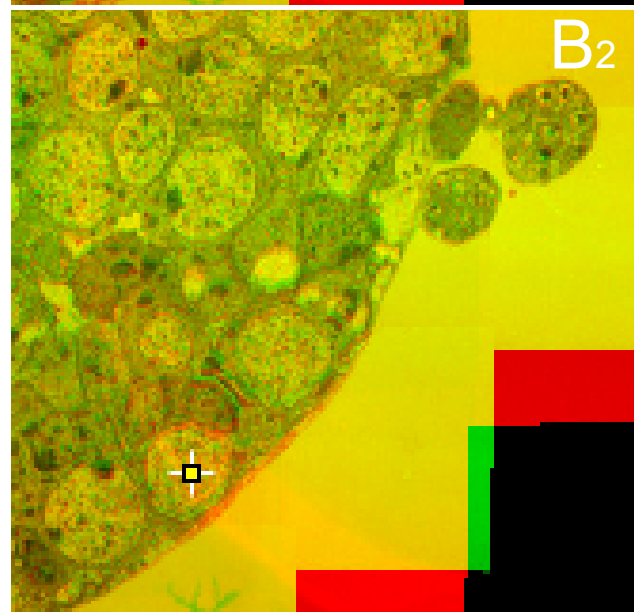

Supplement: Figure S2 — Manual affine transform of collections of image tiles. A The affine transform mode is used for interactive multi-tile transformations. In conjunction with multi-section visualization (the editable section in green, and the previous, reference section in red–the best overlap in yellow), a section is manually aligned to the previous–a capability most useful for correcting or refining the results of automatic registration algorithms. A2 Enlarged inset, revealing the lack of overlap of the two adjacent sections. Notice near top right how the green section doesn’t overlap with the red section. Three landmarks that define an affine transformation are used to interactively adjust the pose of all tiles in the section. B, B2 After manually dragging the landmark the two sections now overlap more accurately. The transformation is then propagated to subsequent sections to preserve the relative pose of all tiles (see menu snapshot in A). (PDF) [file pone.0038011.s002.pdf]

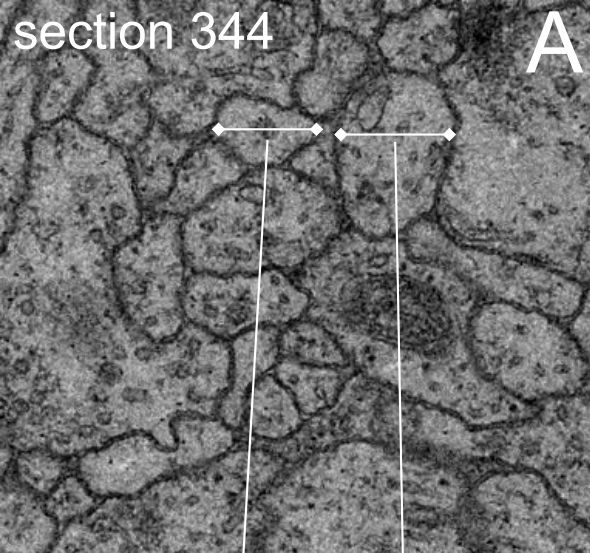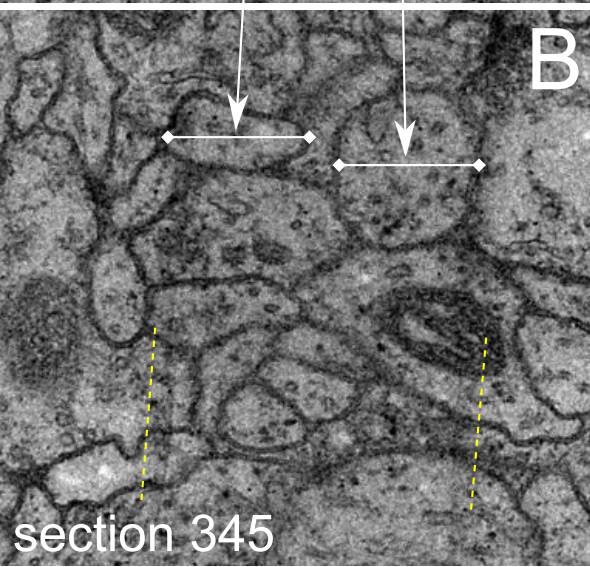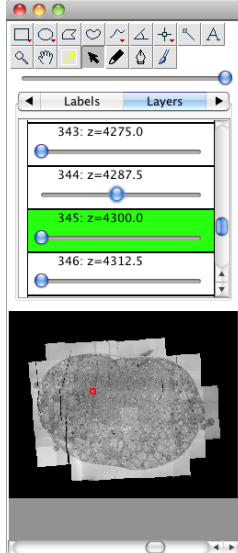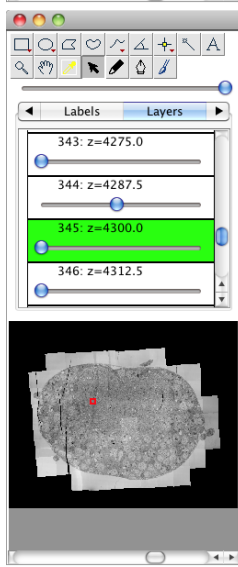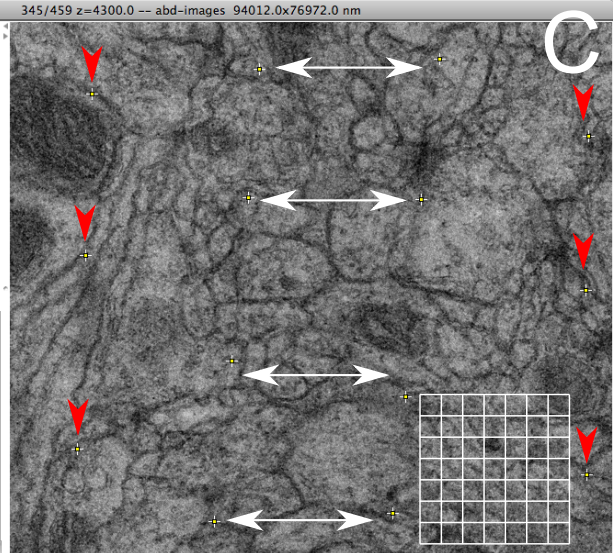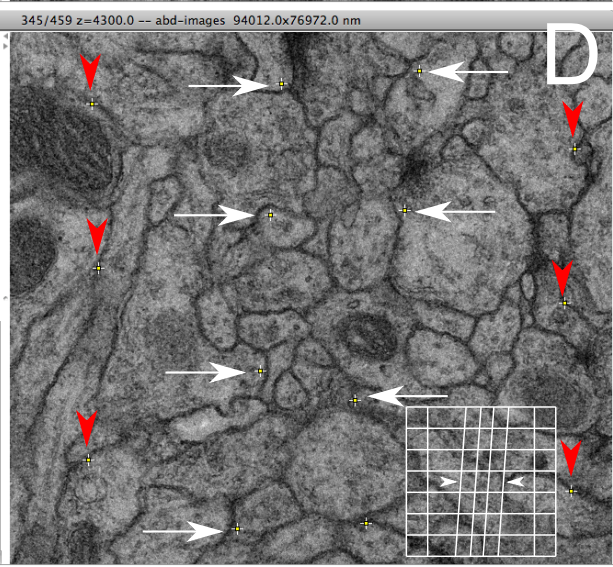

Supplement: Figure S3 — Manual non-linear transform of collections of image tiles for fine cross-section alignment. A,B Two consecutive sections numbered 344 and 345 present an artefactual stretch, as indicated by the widening of the marked profiles (in white). C,D The manual non-linear transformation mode is used here in conjunction with the transparent section overlay (notice the slider above the green panel in C) to reveal the local misalignment. The inset in C,D indicates the local transformation performed by dragging numerous landmarks. (PDF) [file pone.0038011.s003.pdf]

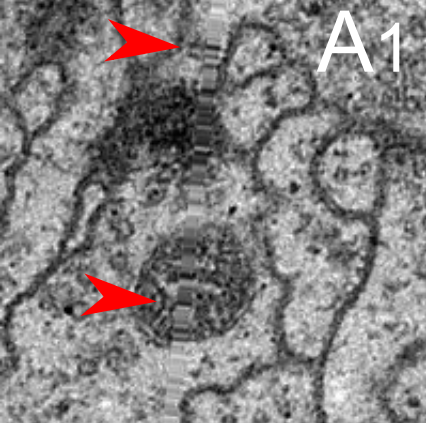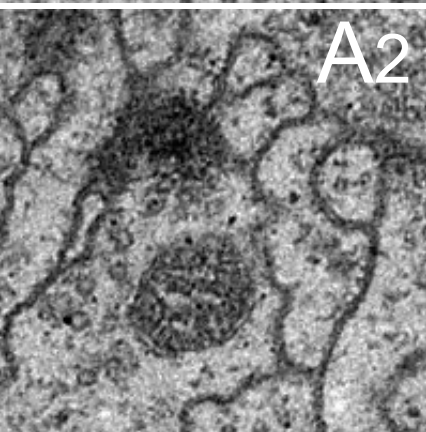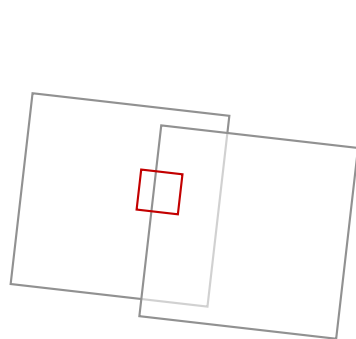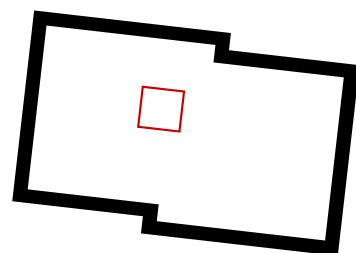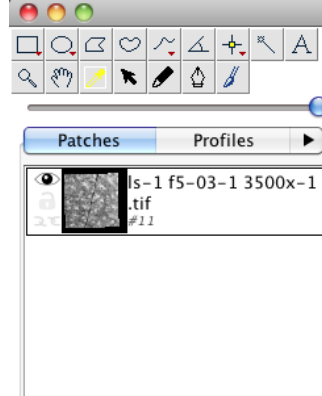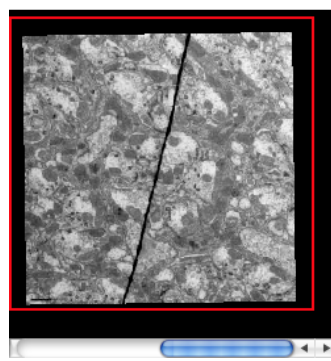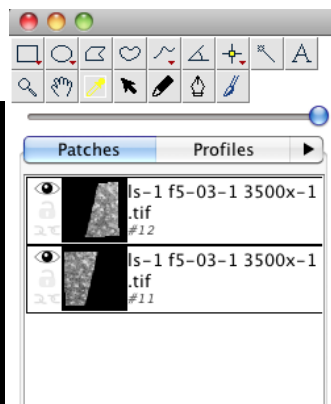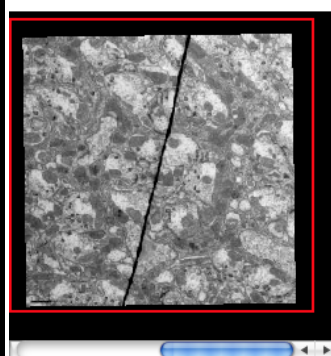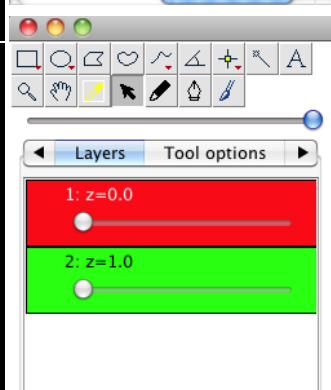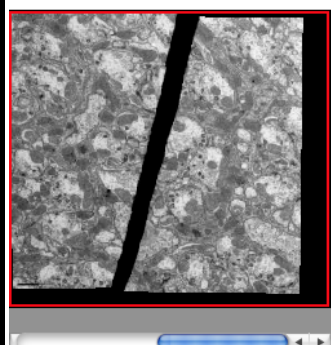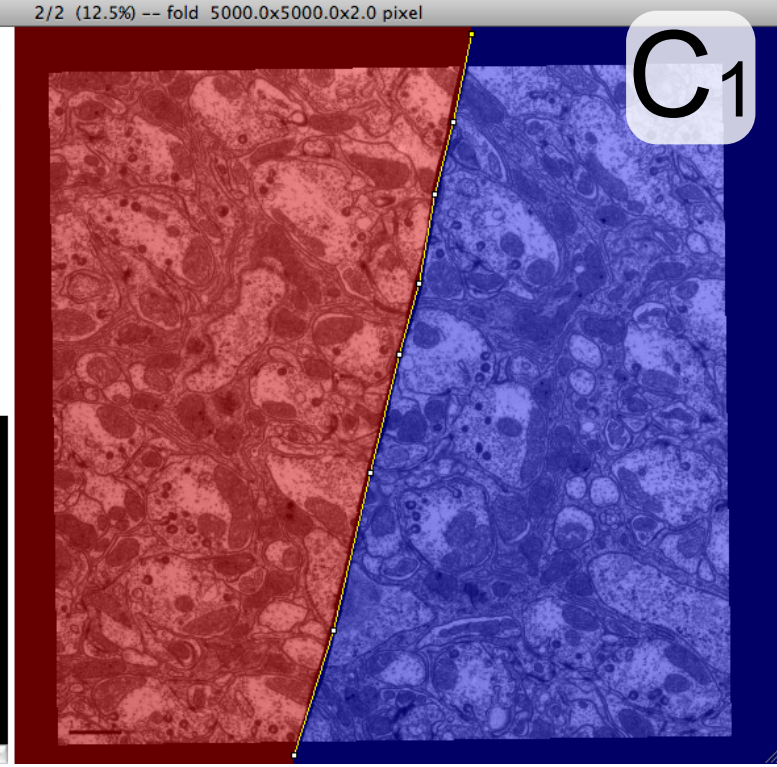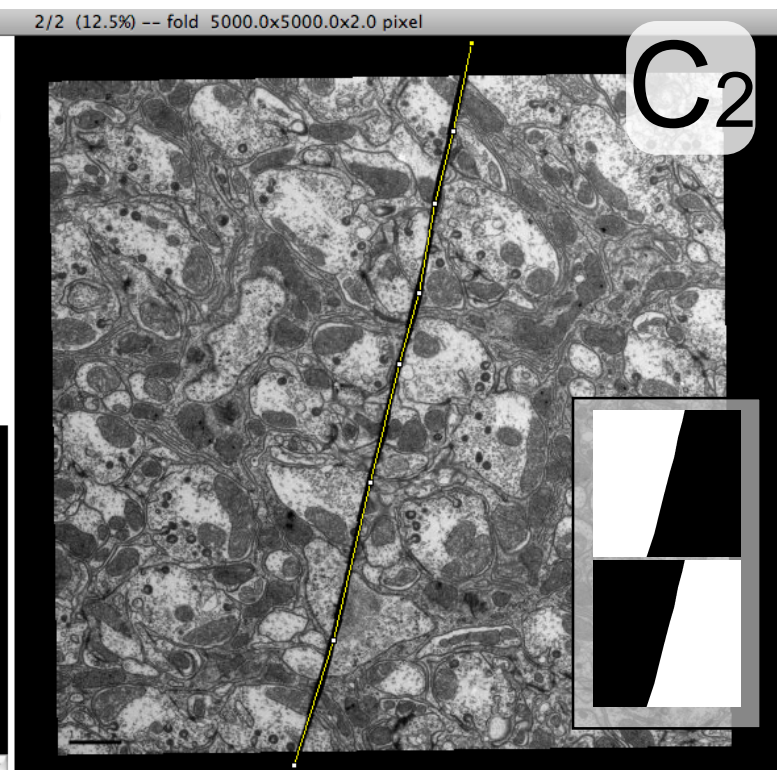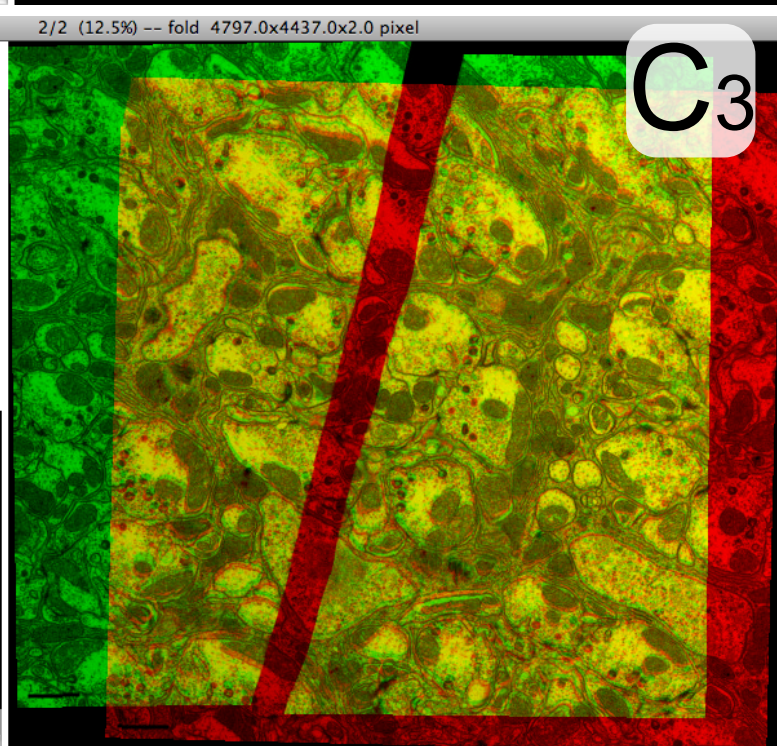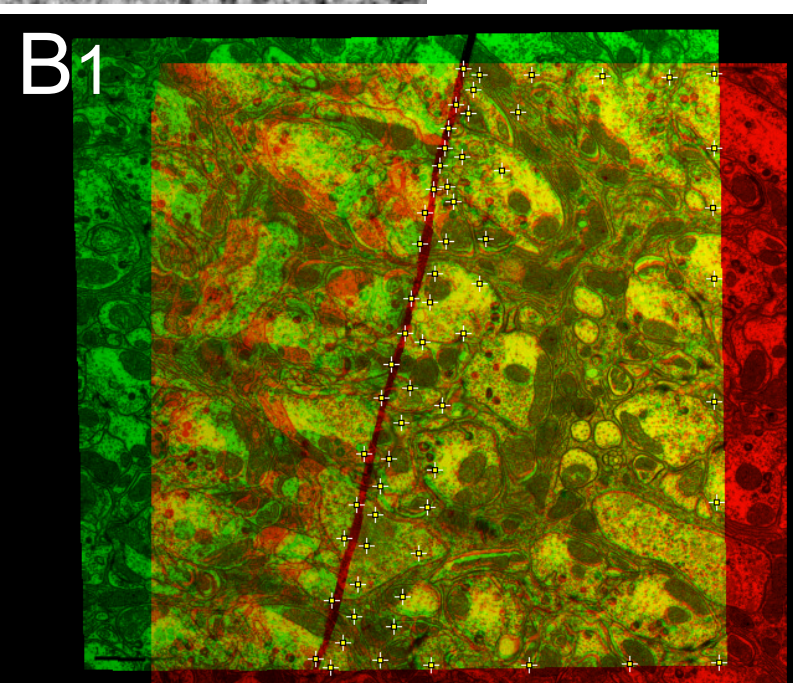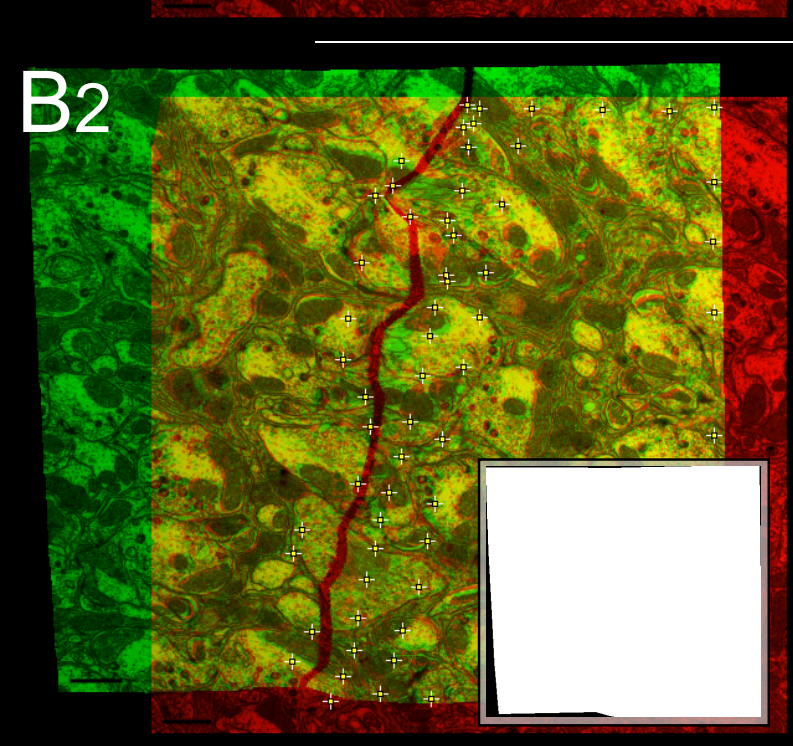

Supplement: Figure S4 — Expressing image transformations without duplicating the original images by using alpha masks. Duplicating images has a huge cost in data storage which TrakEM2 avoids by using highly compressible alpha masks and precomputed mipmaps stored with lossy compression. A Images present borders which are apparent when overlapping (red arrowheads). An alpha mask with zero values for the borders (see adjacent cartoon) removes the border from the field of view. A1 and A2 images show the rectangular region marked in red in the cartoons. B Manual non-linear transformations before (A1) and after (A2) corrects a section fold in an image tile. Inset, the alpha mask of the corrected tile. C Alternatively, the manual image splitting mode cuts image tiles in two or more parts using a polygonal line (C1), so that each half is now an independent Patch object that represents a tile, each relying on the original image but with a different alpha mask (inset in C2). Rigid image registration may now proceed, visualized in C3 by overlaying two consecutive sections. Data in B and C courtesy of Ian Meinertzhagen, Dalhousie University (Canada). (PDF) [file pone.0038011.s004.pdf]

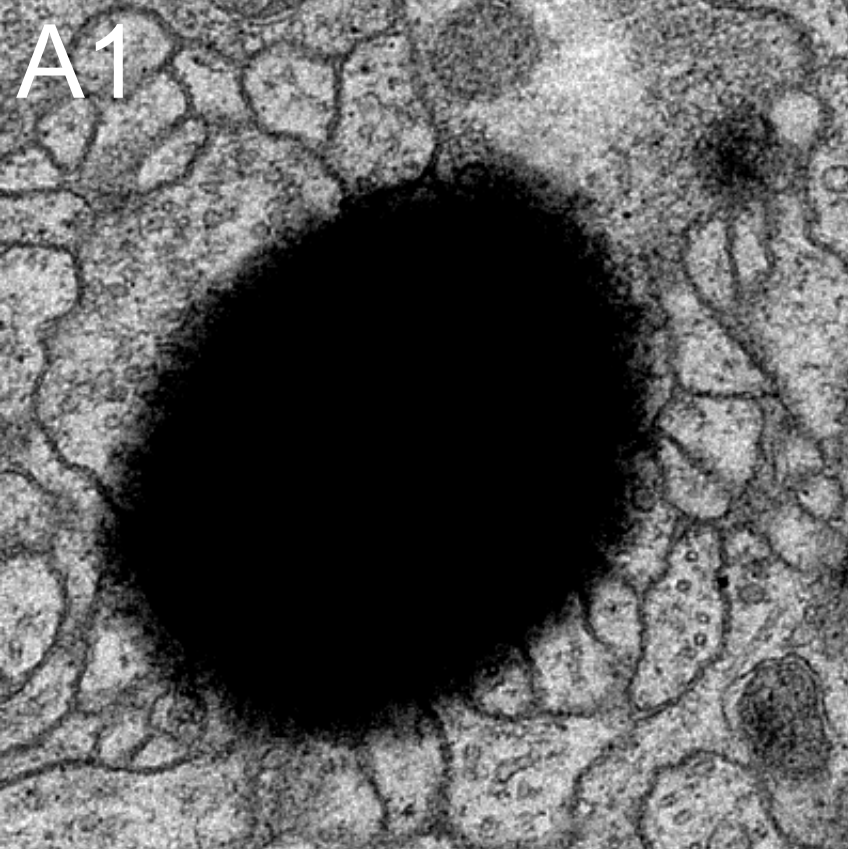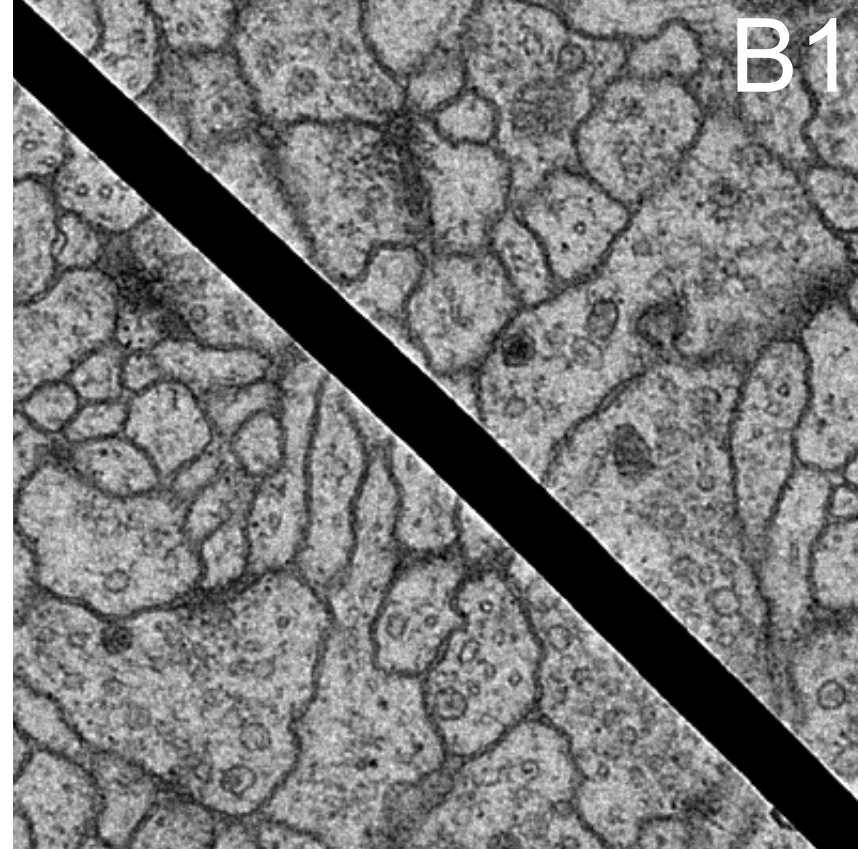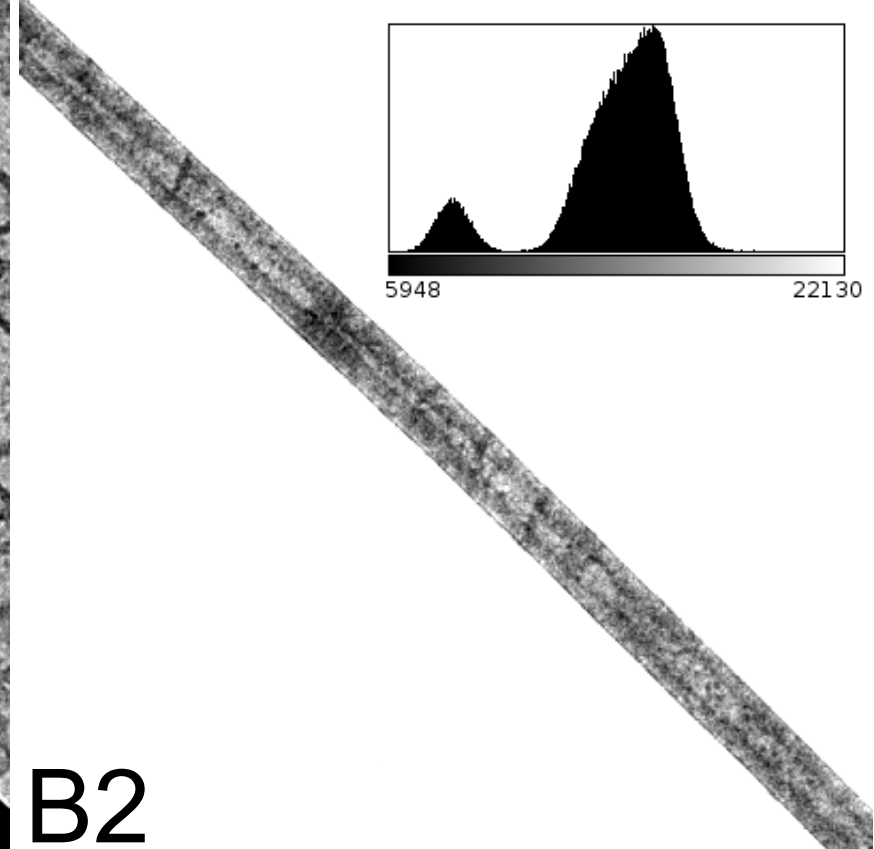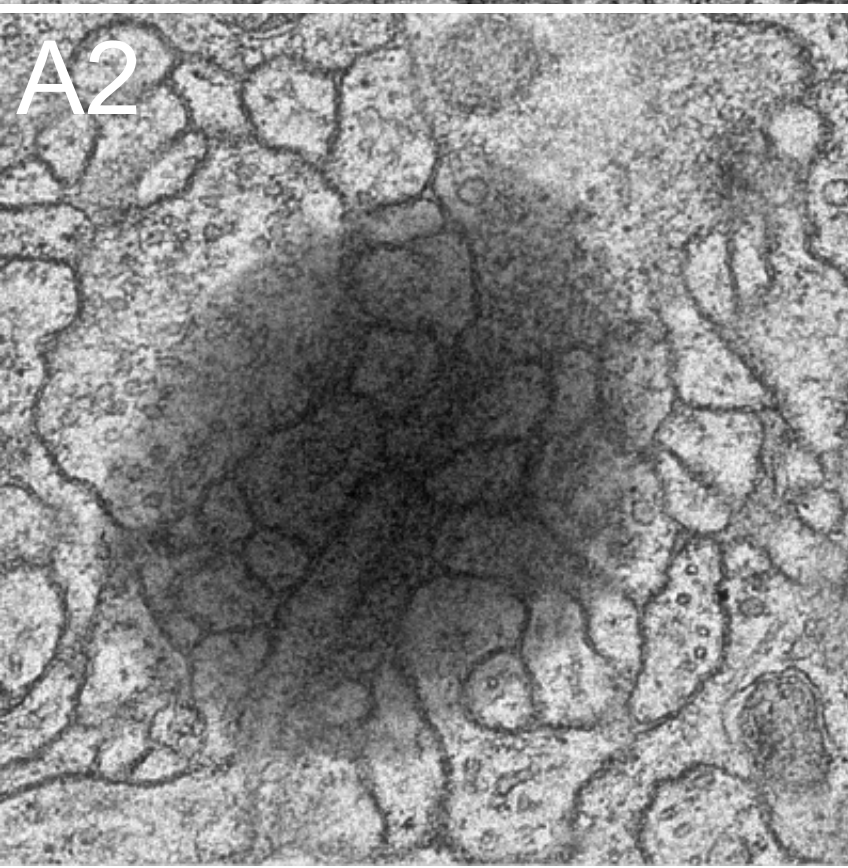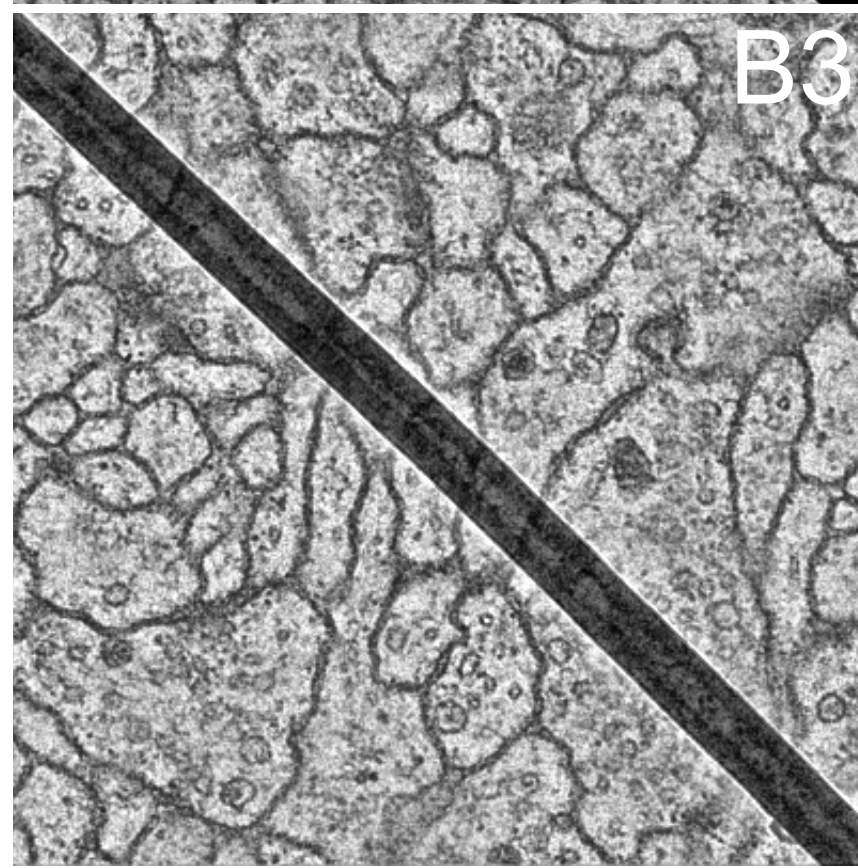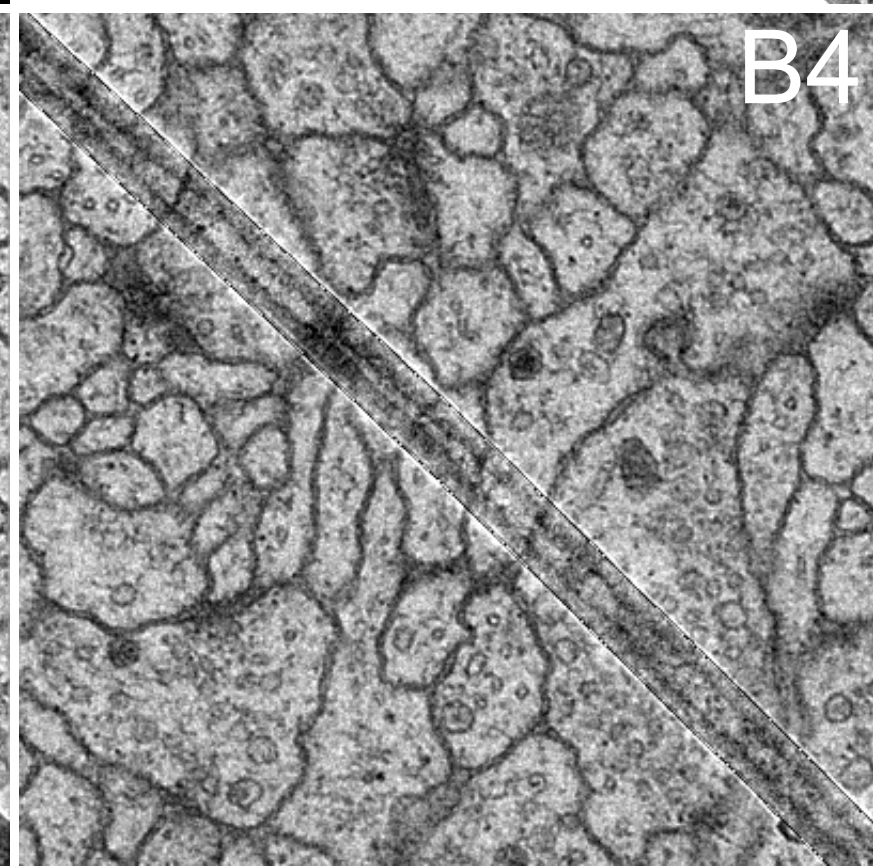

Supplement: Figure S5 — Correctable noise on EM images. A1, A2 A large blob occludes information on an EM image when the display range is adjusted for the whole image (A1), but reveals its content when CLAHE is applied (A2). B1-4 A support-film fold generates a dark band (B1) whose content is discernible at a lower value region of the histogram (inset in B2). Applying CLAHE with a small window partially solves the problem (B3) but composing the image from both ranges restores it best (B4). (PDF) [file pone.0038011.s005.pdf]

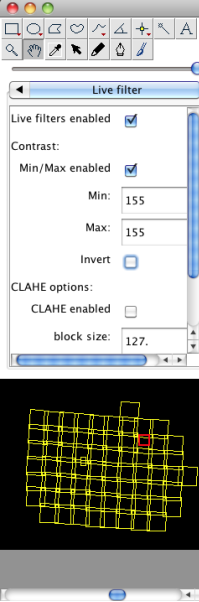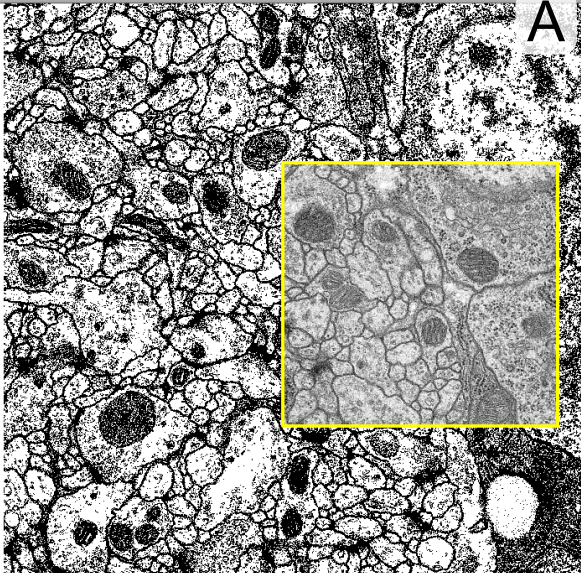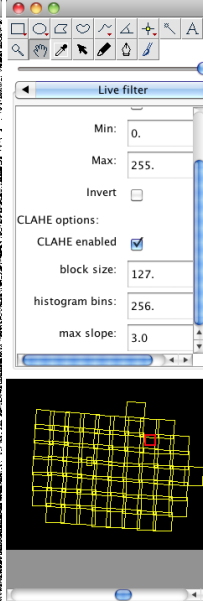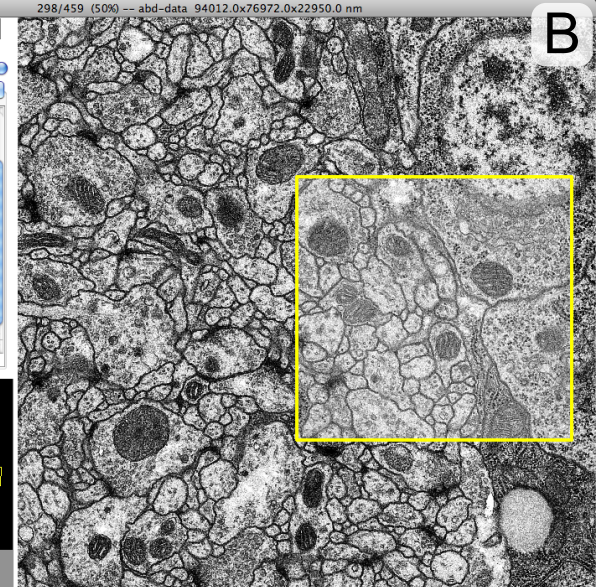

Supplement: Figure S6 — On-the-fly processing of the field of view for enhanced contrast. The live filter tab of the display offers a few filters, to adjust A the display range; invert the image (not shown) or B CLAHE. Yellow rectangle indicates the original view without filters. (PDF) [file pone.0038011.s006.pdf]

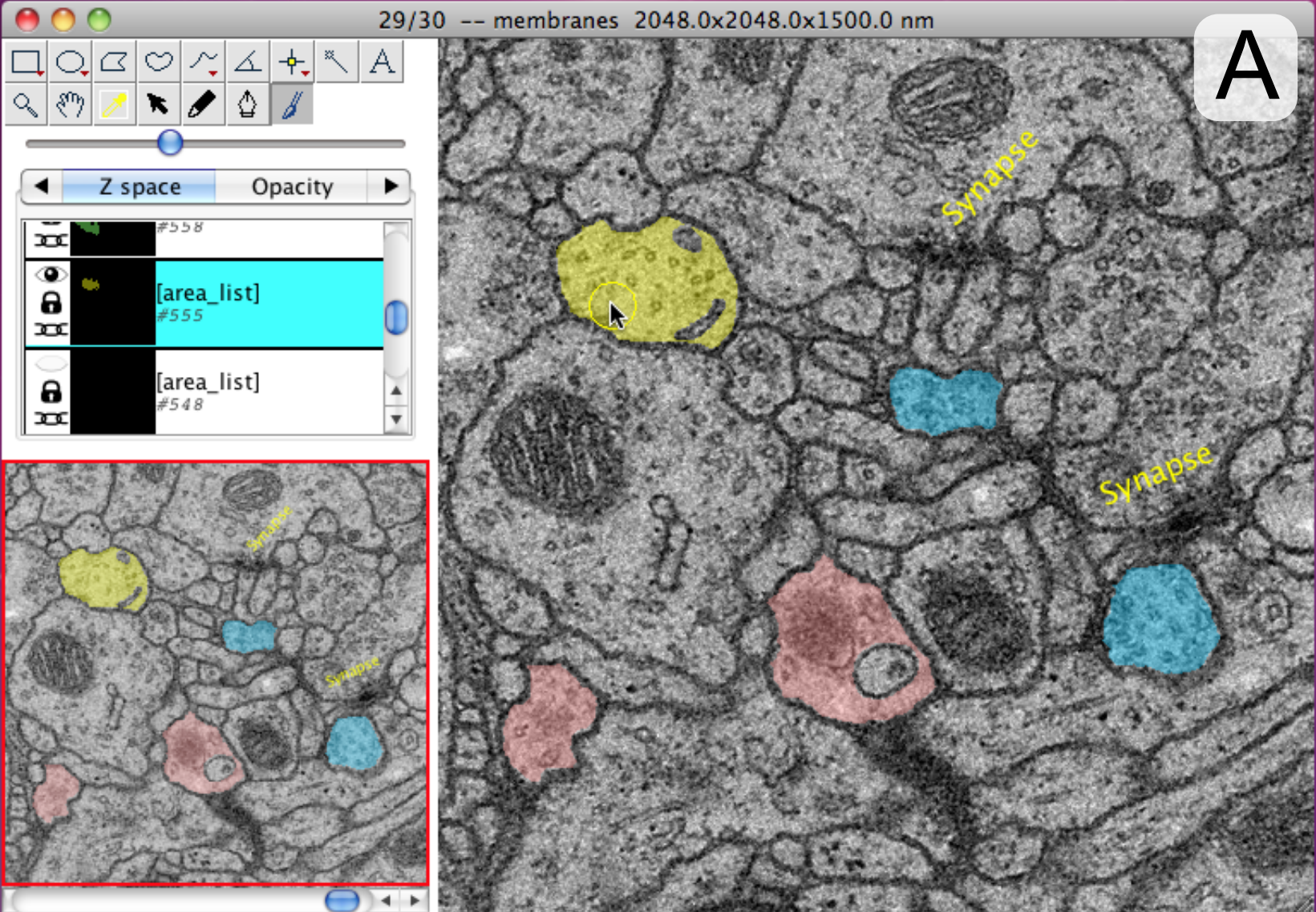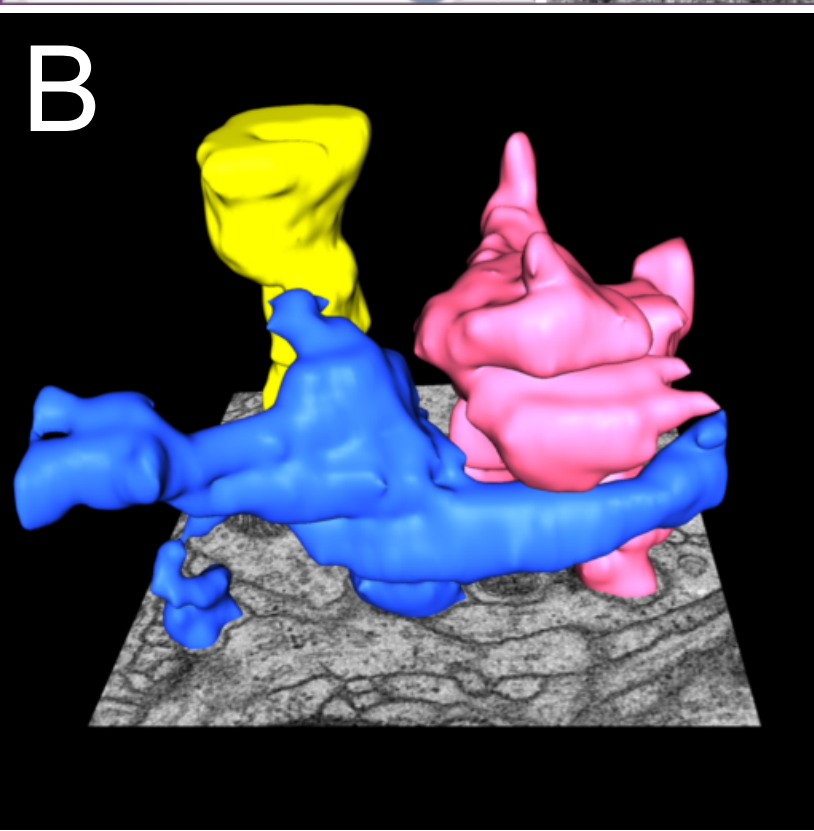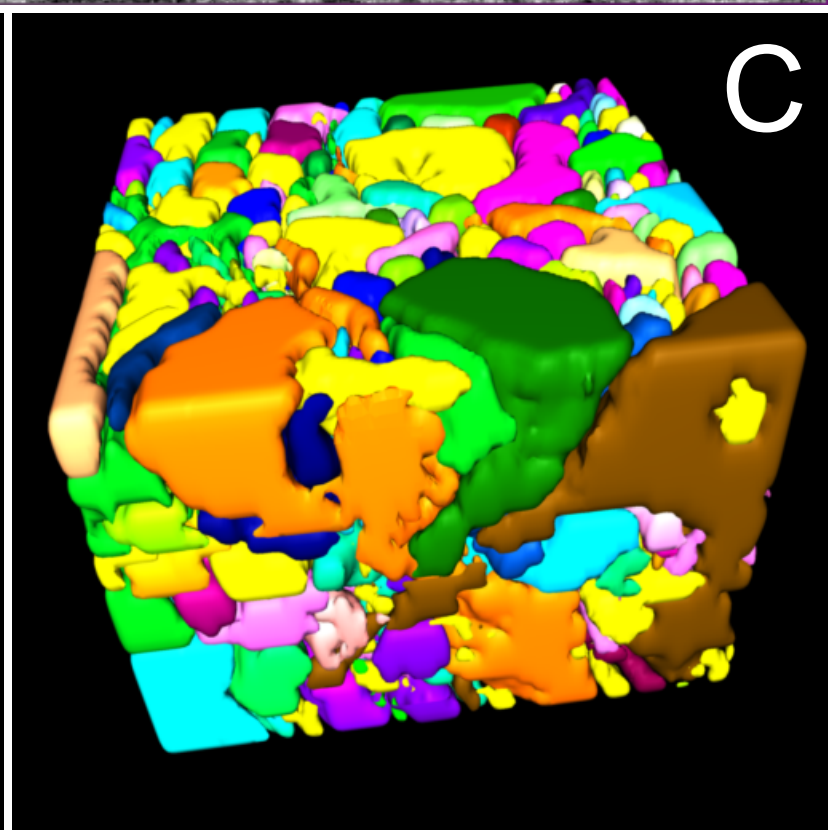

Supplement: Figure S7 — Volumetric reconstruction with series of complex 2d areas or “area lists”. The “Z space” tab lists all segmentation objects that exist in 3d. A With the brush tool, a selected “area list” instance is painted in yellow (notice the mouse pointer with circle), labeling the sectioned profile of a neuron. The selected object (listed in the cyan panel) may be visible or hidden, locked, or linked to the underlying images. B Labeled meshes are rendered in 3d by generating a mesh of triangles with marching cubes. C Dense reconstruction of a cube of neuropil. (PDF) [file pone.0038011.s007.pdf]

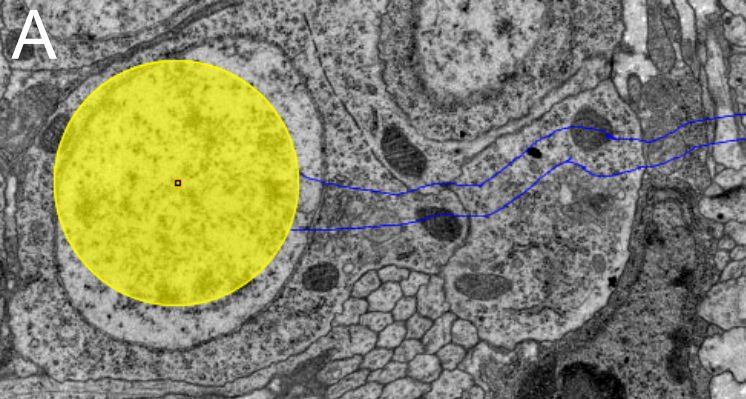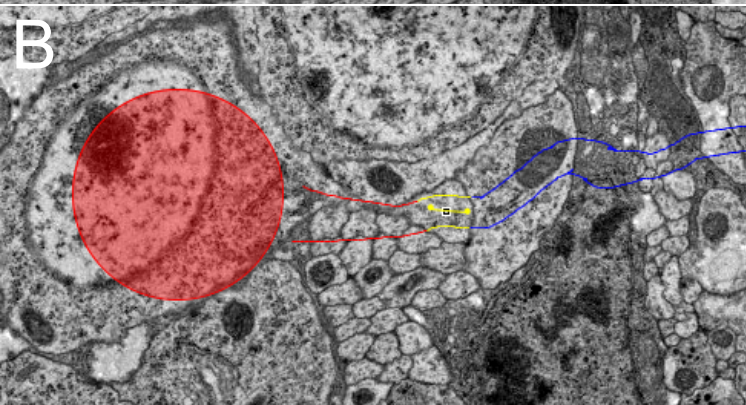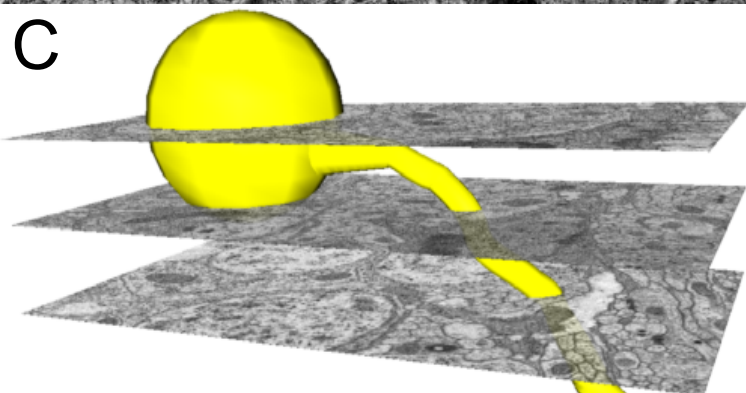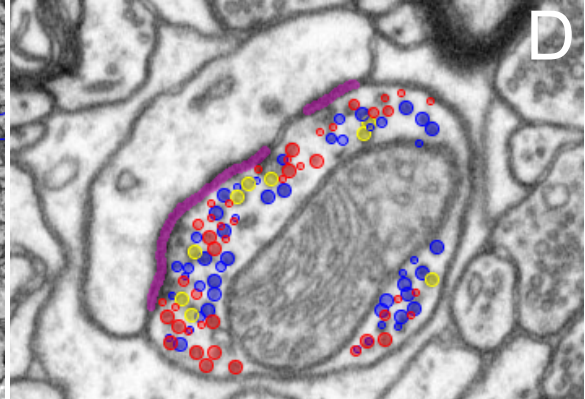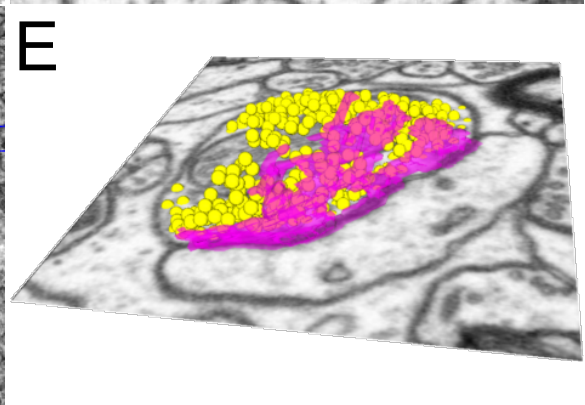

**Ball results**

| File | Edit  | Font |       |         |         |     |        |
|------|-------|------|-------|---------|---------|-----|--------|
|      | units | id   | index | x       | y       | z   | radius |
| 471  | nm    | 160  | 471   | 5105.49 | 4777.42 | 30  | 20.69  |
| 472  | nm    | 160  | 472   | 4735.49 | 4582.42 | 205 | 20.69  |
| 473  | nm    | 160  | 473   | 4805.49 | 4307.42 | 120 | 20.69  |
| 474  | nm    | 160  | 474   | 4885.49 | 4327.42 | 120 | 20.69  |
| 475  | nm    | 160  | 475   | 4720.49 | 4297.42 | 55  | 20.69  |
| 476  | nm    | 160  | 476   | 4710.49 | 4362.42 | 55  | 20.69  |
| 477  | nm    | 160  | 477   | 4365.49 | 4657.42 | 70  | 20.69  |
| 478  | nm    | 160  | 478   | 4400.49 | 4662.42 | 70  | 20.69  |
| 479  | nm    | 160  | 479   | 4435.49 | 4717.42 | 70  | 20.69  |
| 480  | nm    | 160  | 480   | 4500.49 | 4657.42 | 70  | 20.69  |
| 481  | nm    | 160  | 481   | 4425.49 | 4567.42 | 70  | 20.69  |

**F**

Supplement: Figure S8 — Sketching and quantifying neural tissue with spheres and tubes. A,B Two sections with a “ball” to represent the nucleus and a “pipe” to model the main process of a monopolar insect neuron. The colors indicate relative depth: red means below the current section and blue above. C 3d representation of the “ball” and “pipe” traversing multiple sections. D Usage of “ball” sketching type for quantifying the number of synaptic vesicles. The synaptic cleft is modeled with an “area list”. E 3d representation of the synaptic vesicles and cleft modeled in D. F Results table with the count and position of labeled vesicles. Data in D,E courtesy of Graham Knott, EPFL (Switzerland). (PDF) [file pone.0038011.s008.pdf]
