# Supplementary material for: TrakEM2 Software for Neural Circuit Reconstruction
Source: PLoS One. 2012 Jun 19;7(6):e38011. doi: 10.1371/journal.pone.0038011 (PMC3378562; doi:10.1371/journal.pone.0038011)
Supplement: Figure S9 — Measurements. A Example of a “connector” instance, expressing a synapse between an axon (large profile at lower left with numerous microtubules) whose tree is tagged “presynaptic site”, with numerous terminal dendrites (small target circles, one in red indicating it’s in the previous section). B Measurement of the distances from the root node (the soma, by convention) to all nodes labeled “presynaptic site” like in A. The inset schematizes the measurements (dotted red lines from “root” to “nodes labeled as “pre”). C A double disector is used together with an overlay grid (in green, cell size is one micron) to detect the number of objects appearing new in the next section (objects labeled as little yellow squares, with blue circles for the position of the same object in the next section, if present). The table shows the list of all marked objects. Note how “3″ occurs only once, indicating that it appears new in the next section. See [42] for details on the double disector technique. D The built-in scripting editor in Fiji shows a small python script to extract statistics on the distances of synaptic vesicles (modeled with a “ball”) to a synaptic cleft (modeled with an “area list”), as shown in Supplemental Figure 11 d, e. (PDF) [file pone.0038011.s009.pdf]

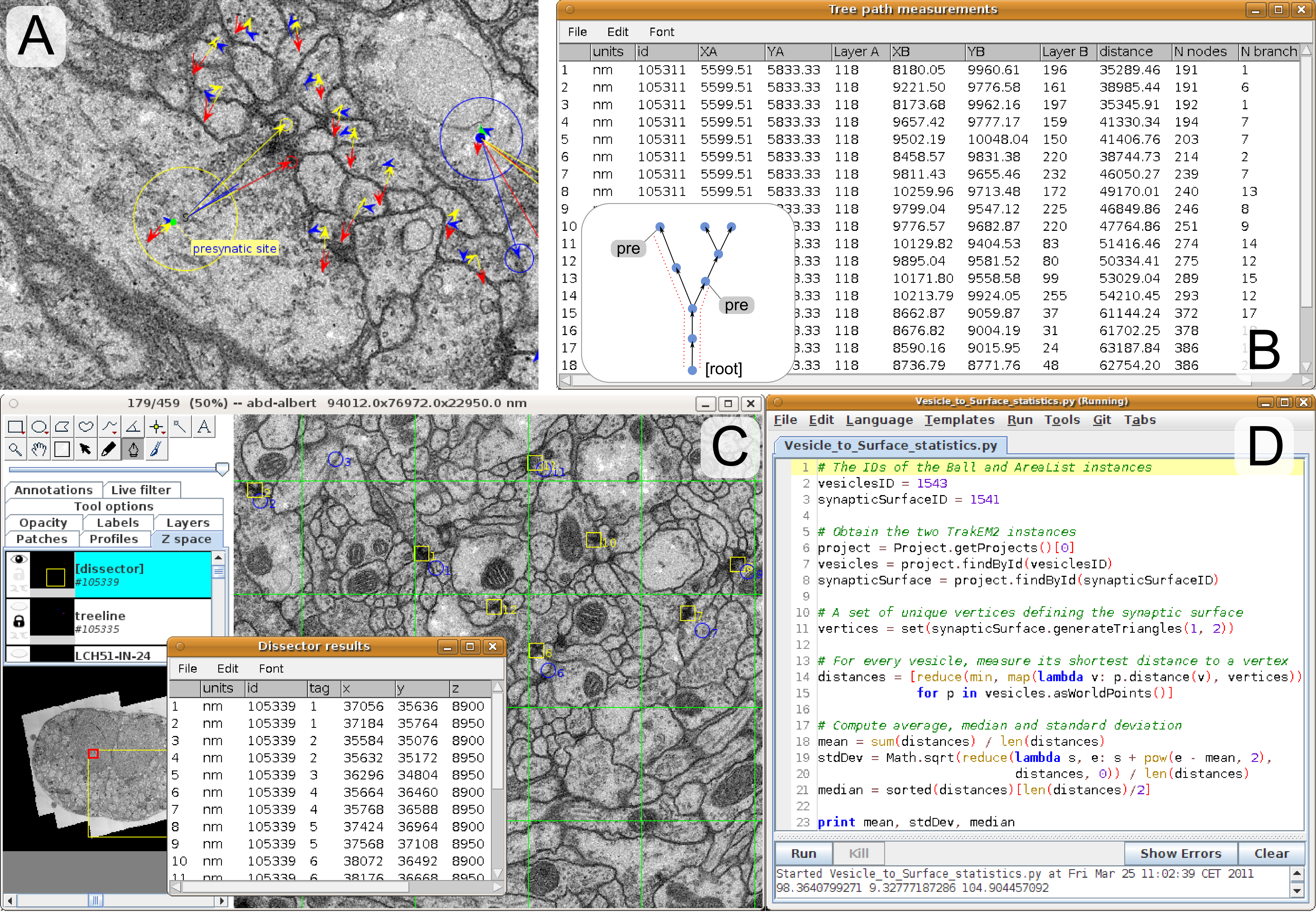
A
presynaptic site
Tree path measurements
File Edit Font
units id XA YA Layer A XB YB Layer B distance N nodes N branch
1 nm 105311 5599.51 5833.33 118 8180.05 9960.61 196 35289.46 191 1
2 nm 105311 5599.51 5833.33 118 9221.50 9776.58 161 38985.44 191 6
3 nm 105311 5599.51 5833.33 118 8173.68 9962.16 197 35345.91 192 1
4 nm 105311 5599.51 5833.33 118 9657.42 9777.17 159 41330.34 194 7
5 nm 105311 5599.51 5833.33 118 9502.19 10048.04 150 41406.76 203 7
6 nm 105311 5599.51 5833.33 118 8458.57 9831.38 220 38744.73 214 2
7 nm 105311 5599.51 5833.33 118 9811.43 9655.46 232 46050.27 239 7
8 nm 105311 5599.51 5833.33 118 10259.96 9713.48 172 49170.01 240 13
9 118 9799.04 9547.12 225 46849.86 246 8
10 118 9776.57 9682.87 220 47764.86 251 9
11 118 10129.82 9404.53 83 51416.46 274 14
12 118 9895.04 9581.52 80 50334.41 275 12
13 118 10171.80 9558.58 99 53029.04 289 15
14 118 10213.79 9924.05 255 54210.45 293 12
15 118 8662.87 9059.87 37 61144.24 372 17
16 118 8676.82 9004.19 31 61702.25 378
17 118 8590.16 9015.95 24 63187.84 386
18 118 8736.79 8771.76 48 62754.20 386
pre
pre
[root]
B
179/459 (50%) -- abd-albert 94012.0x76972.0x22950.0 nm
Annotations Live filter
Tool options
Opacity Labels Layers
Patches Profiles Z space
[dissector]
#105339
treeline
#105335
LCH51-IN-24
C
Dissector results
File Edit Font
units id tag x y z
1 nm 105339 1 37056 35636 8900
2 nm 105339 1 37184 35764 8950
3 nm 105339 2 35584 35076 8900
4 nm 105339 2 35632 35172 8950
5 nm 105339 3 36296 34804 8950
6 nm 105339 4 35664 36460 8900
7 nm 105339 4 35768 36588 8950
8 nm 105339 5 37424 36964 8900
9 nm 105339 5 37568 37108 8950
10 nm 105339 6 38072 36492 8900
Vesicle_to_Surface_statistics.py (Running)
File Edit Language Templates Run Tools Git Tabs
Vesicle_to_Surface_statistics.py
D
# The IDs of the Ball and AreaList instances
vesiclesID = 1543
synapticSurfaceID = 1541
# Obtain the two TrakEM2 instances
project = Project.getProjects()[0]
vesicles = project.findById(vesiclesID)
synapticSurface = project.findById(synapticSurfaceID)
# A set of unique vertices defining the synaptic surface
vertices = set(synapticSurface.generateTriangles(1, 2))
# For every vesicle, measure its shortest distance to a vertex
distances = [reduce(min, map(lambda v: p.distance(v), vertices))
for p in vesicles.asWorldPoints()]
# Compute average, median and standard deviation
mean = sum(distances) / len(distances)
stdDev = Math.sqrt(reduce(lambda s, e: s + pow(e - mean, 2),
distances, 0)) / len(distances)
median = sorted(distances)[len(distances)/2]
print mean, stdDev, median
Run Kill Show Errors Clear
Started Vesicle_to_Surface_statistics.py at Fri Mar 25 11:02:39 CET 2011
98.3640799271 9.32777187286 104.904457092
