# Supplementary material for: TrakEM2 Software for Neural Circuit Reconstruction
Source: PLoS One. 2012 Jun 19;7(6):e38011. doi: 10.1371/journal.pone.0038011 (PMC3378562; doi:10.1371/journal.pone.0038011)
Supplement: Text S1 — Supplemental Text containing detailed information on various aspects of the TrakEM2 software, including image registration, dealing with noise, alpha masks, manual segmentation with areas, balls and pipe objects, and measurements. (PDF) [file pone.0038011.s010.pdf]

1 Supplemental Text

1.1 Browsing large serial EM image sets

TrakEM2 is designed to represent a series of planar sections, each of which composed of an arbitrary number of image tiles. The number of sections and the number of tiles per section are both limited at 2^{31}, the highest signed integer that can be represented with the 4 byte word size of current desktop computers. Each image tile represents a particular image file that resides on the local computer, or on a remote file or web server. The system automatically manages loading, caching and unloading images from their corresponding files.

Many images may be required to cover one tissue section. Each image is encapsulated in a 'tile' that provides methods and data for manipulating it. The images themselves are presented in a two-dimensional software canvas, the *display* (Figure 1e of main text). The location of each image is controlled by its tile. Each image tile is manipulable as an independent unit both by the human operator or programmatically, and has persistent properties such as affine and non-linear transformation, transparency value and alpha mask, among others. Arbitrary properties may be stored as key-value pairs for each tile. A variety of image types (8-bit, 16-bit, 32-bit, 8-bit color, RGB) may coexist within the same section and display. Image tiles overlap following a specific, editable order, and are composed into the field of view with consideration of the alpha channel, alpha value, and composite mode (add, multiply, subtract, difference, and YcbCr color) of each tile (Figure 1d of main text).

Images are stored in tiles in their original source data form. These tiles are mapped to the display by concatenating their series of locally stored transformations. A collection of general-purpose coordinate transformations (affine or non-linear) enable arbitrary deformation, e.g. to correct for lens distortions, or heat-induced non-linear deformations, for examples. An affine transformation positions the resulting image into the 2d coordinate space of the section. A translation and scaling transformation projects the images into the coordinate space of the field of view.

For fast visualization of arbitrary fields of view, the image is rendered concatenating the list of coordinate transformations and stored in disk as an image pyramid, which is composed of pre-scaled versions of the image called *mipmaps* (or MIP maps, where MIP stands for *multum in parvo* Williams, 1983). These are stored with lossy or lossless compression to relieve the already large storage space requirements of the raw data, and they are loaded on demand with a caching strategy. Each section uses a quad-tree strategy for fast lookup of tiles that intersect the field of view.

The EM automatic image acquisition software Leginon (Suloway et al., 2005) records the slice and coordinates of each tile. This information is used for generating a 4-column text file, with data for

the file path, x and y coordinates, and slice index. This minimal information is sufficient for importing into TrakEM2 all image tiles and presenting them in a virtual canvas for their subsequent manipulation. Other methods for importing individual image files and complete montages contained in file series are available by drag and drop of images and folders, respectively. Direct programmatic access with any of the languages supported by the Java Virtual Machine (JVM), such as python, is available, providing the means to extend the functionality of TrakEM2 for any special-purpose application.

1.2 Assembling the volume with automatic registration of image tiles

The work flow for neuronal reconstruction from ssTEM begins by assembling the image volume from a large number of image tiles (hundreds of thousands). These images have been acquired manually or automatically, for example with Leginon (Suloway et al., 2005) or SerialEM Mastronarde (2005). Automated image registration algorithms face obstacles such as noise that occludes image data, section folds, missing sections, lens distortion, and a variety of physically- and heat-induced non-linear deformations. Calculating the correct alignment across all sections is difficult and prone to artefactual deformations.

In order to cope with these difficulties, TrakEM2 provides a flexible set of tools for automatic image alignment and deformation correction both within and across section montages. The tools split into two categories: landmark based and intensity based. Landmark based techniques extract corresponding landmarks in two images using the Scale-Invariant Feature Transform (SIFT; Lowe, 2004). SIFT interest points are blob-like structures in an image. For each interest point, a scale and rotation-invariant descriptor is extracted such that correspondences candidates between two images can be identified by matching the descriptors of all extracted features. For overlapping images, the set of correspondence candidates will contain both true and false matches. For non-overlapping images, there are only false matches. Such false matches are rejected using a combination of the Random Sample Consensus (RANSAC; Fischler and Bolles, 1981) and a robust regression filter (Saalfeld et al., 2010) delivering the largest set of correspondence points that support a common transformation up to an approximately normal distributed transfer error. For non-overlapping images, this set is empty which is used to reveal non-initialized montages and to identify disconnected or empty images automatically.

Digital EM images often show a static background texture that is an uncompensated artifact of fiber optics in the scintillator that transforms incoming transmitted electrons into photons for the camera to capture. TrakEM2 is able to ignore such static background texture during alignment by rejecting the largest set of true matches if the supported model is the identity transformation. Instead, the second largest set is used if available.

Automatically extracted landmark correspondences are used for fast series alignment, section

montaging and to automatically correct for the EM magnetic lens deformation using redundantly overlapping calibration montages (Kaynig et al., 2010a).

The most sophisticated landmark based alignment method establishes correspondences for each tile not only to overlapping tiles within the same section but as well to all overlapping tiles in adjacent sections. Finally, a global optimizer estimates an independent rigid transformation for each tile such that the sum of all square landmark correspondence displacements is minimized (Saalfeld et al., 2010). If the result is directly applied as a rigid transformation to each tile, section montages will appear imperfectly stitched because displacements within a section compensate for cross-section distortion. Instead, for each montage, a smooth control-point based non-linear transformation is created that maps all tile center points from the independent montage into the desired location. This transformation smoothly warps each montage into global series alignment.

This method is also capable of registering very sparse representations of a neural arbor, such as multiple non-overlapping images in each section which are centered on the dendrites or axons of interest. The lack of relationships between images in the same section is offset by the graph of image relationships across 3d space, which is used to compute the optimal pose of each image in the volume.

Except for the optimizer, all operations proceed in parallel for maximum performance on modern multi-core chips. The registration of 33,000 image tiles (2048x2048 16-bit px) spread over 458 sections requires 5 days in a computer with a 4-cores 2.4 Ghz CPU and 4 Gb of RAM.

For best performance, sections are montaged automatically and independently beforehand. This is efficient given that the original image tile is neither modified nor duplicated, but a mere affine transform is recorded for its pose in space.

Usually the approximate location of each tile is known either from the index (image tiles are acquired in order) or from the stage position information contained in the file's metadata. Intra-section montaging is thus reduced from an all-to-all search, to a search for correspondences between tiles known to be overlapping. Then a low-resolution snapshot of consecutive sections is used to estimate a rough alignment using a rigid transform. In this manner, registering all sections is reduced to a search of correspondences between image tiles that overlap within or across sections. This strategy reduces immensely the number of operations to compute: From $3 \times$sn-1 per tile (intrasection and with previous and next sections, where sn is the number of tiles in a section) to typically 16 per tile.

Serial section EM data sets may grow over many months or years. It is usually desirable to begin analysis when only a subset of sections has been imaged. This raises the problem of re-registering the existing registered volume along with annotations and any reconstructed elements such as neurons, as the data set expands. TrakEM2 stores the extracted SIFT features and their discovered correspondences

of the current model in temporary serialized data files. As new sections are inserted or appended, the automatic registration can make use of these stored features and correspondences. This approach greatly reduces computation time at the cost of marginal storage space. The manually or automatically reconstructed neurons and other elements are transferred to the updated model, after transformation of their underlying image tiles.

TrakEM2 offers three intensity based methods for image alignment. Firstly, phase-correlation based montaging (adapted from Preibisch et al. (2009), which is a very fast method for montaging a single section with translation-only transformations. The method is best used when there is prior information about the approximate position of each tile. Tiled fluorescence images, which are generally sparse and for which SIFT features can be extracted only with difficulty, are best montaged using the phase correlation method. Secondly, section series can be aligned sequentially using cubic B-splines. These are pre-seeded with a rigid or affine transformation (computed from SIFT correspondences) that estimates a rough initial alignment. The method applies the software library bUnwarpJ (Arganda-Carreras et al., 2006), and is useful for short series where artifacts generated by non-linear registration do not pose a significant problem. Finally, TrakEM2 incorporates a method for elastic montaging and series alignment (Saalfeld et al., submitted to Nature Methods). In this method, images are modelled as elastic sheets using a particle-spring system. Corresponding locations in overlapping images are estimated using block matching and connected by zero-length springs. Relaxing the whole system creates a set of aligned images with the required non-rigid deformation being equally distributed among all images.

1.3 Manually correcting automatic image registration with affine and non-linear transformations

Automatic image registration methods may fail to achieve sufficient results in the presence of multiple sources of noise or missing data. TrakEM2 enables to manually and programmatically manipulate every image tile in the data set. Both the non-linear and affine transformations of a tile or a group of tiles in a section are adjustable from the graphical interface (Fig. S2, S3), and means are provided to propagate the resulting transformation to subsequent sections. In this manner, we address a frequent problem in serial section EM: The presence of gaps in the series, be it missing sections or noise in a section that make inter-section registration difficult. In such case, the serial section set is automatically partitioned into independent continuous subsets, expressed as independent graphs of connected image tiles. The sections of each subset are registered automatically and independently of other subsets. Thereafter the last section of one subset and the first section of the next are registered manually.

For the purpose of manual multi-section registration, the display of TrakEM2 is able to present

simultaneously the montages corresponding to multiple sections. There are several strategies. (1) Render up to three sections, each as a color channel in an RGB image (red, green and blue); regions that correspond across sections appear in yellow when two sections are presented one in red and the other in green (Fig. S2). (2) Render any number of sections transparent, with section-wise composite strategies such as add, subtract, multiply, difference, and color (YCbCr). The *difference* composite mode is most practical for visually evaluating the correctness of the registration (Fig. S1 a, b).

Section folds complicate the alignment of series of sections. A fold on the otherwise flat 50 nm-thick section appears as a blackened stripe that swallows part of the surface and leads to missing data. Stretching the folded area is possible with an interactive non-linear registration method (Fig. S4 b). Alternatively, the image tiles under the fold may be split at the fold line, allowing the halved tiles to move and stretch independently for optimal registration with adjacent sections (Fig. S4 c). A similar method is applicable to dilated bands (Fig. S3).

1.4 Image adjustment

Raw images from the electron microscope must be adjusted for both processing and visualization purposes. Raw images have uneven illumination, low contrast, and areas with extreme differential contrast such as those under the shadow of a fold in the support film (Fig. S5 b). Some kinds of noise (such as precipitated uranyl acetate) can be largely removed by local contrast adjustment (Fig. S5 a). Adjusted images are not only easier for the human observer, but also crucial for the correct operation of key algorithms. For example, feature extraction with SIFT (Lowe, 2004) deliberately works in the specified contrast range of an adjusted image by which the algorithm can be directed to focus on desired texture while ignoring background or noise.

TrakEM2 offers a variety of automatic and manual image adjustment tools. The minimum and maximum values of the display range of an image tile or group of tiles is adjustable. The histogram of one or more tiles can be equalized or normalized, either tile-wise or by incorporating the statistics of a group of tiles. The most powerful manipulation method is with preprocessor scripts, which are arbitrary programs that modify the image data after it is loaded from the file but before TrakEM2 handles the contained image. These scripts are written in python, javascript or beanshell, three languages implemented for the JVM, and have complete access to all included software libraries. A common setup is to adjust uneven illumination by correcting with a bright-field image (that is, an image of the background, sometimes computed from the image itself by applying a median filter with a radius of half the image width), or to perform contrast limited adaptive histogram equalization (CLAHE, Zuiderveld, 1994). The application of all the above methods alters the precomputed image pyramid of each tile (the mipmaps)

and the properties of each image tile, but the original images remain unchanged.

TrakEM2 also offers live filters, that process the screen image that shows the field of view and not the underlying image tiles or their mipmaps on the fly. Live filters have the advantage of being very fast and light-weight, and are sufficient in many occasions. Included live filters are display range adjustment, inverting the image and CLAHE (Zuiderveld, 1994; Fig. S6).

Raw images usually have undesirable borders. These are smeared insets, an artifact of the CCD chip of the EM camera. Cropping the complete collection of original images only to remove the border would result in duplicating the size of the data set, or destroying original images–both unacceptable practices. TrakEM2 offers automated methods to remove these borders by applying an alpha mask to each image tile, which specifies areas of the image that are rendered with arbitrary transparency values (Fig. S4). These masks are compressed with a lossless algorithm such as ZIP and usually take up less than 1/1000 of the image file size. Beyond masking borders, alpha masks can be set manually or programmatically and are useful to express non-linear transformations in images without having to crop the borders to the minimally included rectangle (Fig. S4 b), and also to split images into two or more independent tiles for the purpose of independent registration (Fig. S4 c). Alpha masks are included in the precomputed mipmap images for best performance.

1.5 Image segmentation for 3d object reconstruction

Imaging and registering serial sections is only the first step towards the reconstruction of neuronal circuitry embedded in a set of serial sections. Each individual structure of interest, be it a neuron, a synapse, a glial cell, a vessel, or parts thereof, may be reconstructed precisely or coarsely using different tools offered by TrakEM2. The most accurate reconstruction is volumetric and consists in labeling areas with a paint brush in every section where the structure appears. For example, the cross section of a dendrite may be brushed in a specific color over multiple sections. The calibrated dimensions of the section specify a volume for that 2d area; the concatenation of areas from all sections with an algorithm such as marching cubes (Lorensen and Cline, 1987) results in a 3d mesh model. TrakEM2 uses the "3D Viewer" library for 3d visualization and mesh operations (Schmid et al., 2010; Fig. S7).

Accurately labeling areas by hand is a very time-consuming task. TrakEM2 provides three semi-automated methods: (1) fast marching level sets, which grow an active contour from a seed point (Sethian, 1996); (2) a lasso tool that grows an area interactively according to pixel value intensities; and (3) a magic wand that is most useful for homogeneous areas such as structures found in laser-scanning microscopy images. Fully automated methods are currently the subject of intensive research. The leading strategies employ machine learning algorithms such as Random Forest (Breiman, 2001), Support

Vector Machines (Cortes and Vapnik, 1995) or Convolutional Neural Networks (LeCun et al., 1989) that are trained to classify pixels as belonging to user-defined labels by inspecting a vector of features for each pixel (Kaynig et al., 2010c,b; Turaga et al., 2010). These features are statistical measurements taken for a defined area centered at the pixel (for a detailed explanation see Jain et al., 2010), including edge detectors, difference of Gaussian values, Gabor filters and many simpler ones such as minimum, average and maximum pixel intensity values. *Fiji*, the open source image processing application that hosts TrakEM2, offers an implementation of these machine learning approaches in the plugin *Trainable Segmentation*. Newer impressive tools like *ilastik* (Sommer et al., 2011) perform similar operations in three dimensions for isotropic volumes. The results of applying machine learning-based segmentation are image volumes that contain regions defined by unique pixel values. These are imported into TrakEM2 and converted there into editable vector graphics objects that are overlaid on the image tiles.

A neuronal arbor is represented across the serial section set as a series of 2d areas. In the software Amira (AmiraVis), these areas are represented by voxels and are not structured. In the software Reconstruct (Fiala, 2005), the areas are structured as a list, at one per section; each area is really a multi-path object that can express topological constructs such as holes and islands, which are beyond a simple undivided area. This structure is mimicked by TrakEM2's "area list" segmentation type (Fig. S7 a). Both representations present difficulties when errors are found in the reconstruction. A typical example is that a branch does not belong to the particular neuronal arbor of interest, and it must be split away. Branches traverse the same sections in multiple directions, and thus within the same section different branches are expressed by a single multi-path area object without any explicit relationship with the corresponding area in adjacent sections other than approximate position. The correction is error prone and time consuming. The usual approach is to erase the incorrect branch instead of merely splitting it, incurring in additional labor.

A better segmentation data type would be structured to correspond, topologically, to the structure of interest; in our case neuronal arbors (Figure 2 of main text). TrakEM2 offers the "area tree" data type, which consists of a tree of parent/child nodes where each node hosts a single area (Figure 2e,f of main text). The tree is re-rootable at any node and thus any branch with all its subbranches may be split away with a trivial operation (Figure 2 h-j of main text). Merges are also trivial. Splits and merges are the two necessary operations for correcting automatic neuronal reconstructions (Jain et al., 2010; Chklovskii et al., 2010).

The second aspect of both manual and automatic segmentation in anisotropic EM images is the linkage of 2d areas across sections (Kaynig et al., 2010b; Chklovskii et al., 2010). In all existing image segmentation programs this relationship is expressed as all or nothing. In TrakEM2, the parent/child relationship of nodes in an "area tree" is annotated with a confidence value that expresses the certainty

in the relationship. In the tabular view of the nodes of an ”area tree”, nodes are sortable also by confidence, which enables rapid inspection of all dubious assignments (Figure 2c of main text). With noise, missing sections, and very thin dendrites shadowed away by the thickness of a section (50 nm), the confidence value is useful in expressing the minimally trustable arbor of a neuron, with all potential terminal branches being skeletonized as well but with lower confidence. Some of the branches labeled with a low confidence value may be validated to some extent with confocal microscopy imaging of the same neuron, a comparison possible in stereotyped systems such as *Drosophila* (Cardona et al., 2010); or later on by elimination when the reconstruction of the image volume approaches completion (that is, all possible neurites have been skeletonized). The concept of confidence in the assignment of relationships is also applied to postsynaptic targets of a presynaptic bouton, as represented by instances of the ”connector” data type (see below). This confidence is useful to express potential relationship when the postsynaptic nature of a terminal cannot be fully elucidated (Figure 2h of main text).

1.6 Stick-and-ball models

TrakEM2 supports both fluorescently labeled and EM image volumes. The resolution of laser-scanning microscopy (LSM) is improving with supraresolution techniques (such as STED, Klar et al., 2000; and PALM, Betzig et al., 2006) but these are not yet applicable to image volumes. In the usual case details such as terminal dendritic branches are not resolvable in LSM image volumes. A useful operation is to automatically trace the lowest-order branch of a neuron and then compare it across multiple brains to quantify stereotypy or to elucidate the identity of the neuron relative to a reference database (Cardona et al., 2010). TrakEM2 offers the ”pipe” and ”polyline” data types which are useful for sketching tubular structures with Bézier curves and polylines, respectively (Fig. S8). The automatic tracing tool enclosed in the Simple Neurite Tracer program and library (Longair et al., 2011) is transparently accessible from TrakEM2 for semiautomatic reconstruction of neuronal branches. The results of the semiautomatic tracing are expressed as instances of the ”polyline” data type.

The topology of a neuronal arbor and the location of synapses on it are essential for reconstructing neuronal circuitry. The precise reconstruction of the volume of a neuronal arbor is useful for functional yet not essential for anatomical circuitry reconstruction. The ”area tree” data type offered by TrakEM2 doubles as an area container for volume reconstruction and a skeleton representation of a neuronal arbor (Figure 2e,f of main text). The ”area tree” enables practical approaches such as manually sketching the arbor and then automatically filling in the area of sectioned axons and dendrites, by using each tree node as a seed point for level sets or for picking an area generated by machine learning-based image segmentation methods. The ”tree line” data type uses a radius at each tree node for a stick model

of the arbor (Figure 2 a-c,g of main text).

TrakEM2 also offers a ”ball” data type, which represents a group of spheres. This tool is useful for example for the representation and counting of neuronal nuclei or synaptic vesicles (Fig. S8).

1.7 Annotation

An EM serial section set contains a large number of structures of interest such as neurons and synapses. In the course of the analysis, the human operator or an automated search algorithm identifies numerous structures that must be annotated for future reference. For this purpose, TrakEM2 offers floating text labels, which are searchable with regular expressions.

The manual reconstruction of a neuronal arbor is complicated by its branchiness. The ”area tree” and ”treeline” data types consist of a tree of parent/child nodes, where each node holds spatial coordinates and can be annotated with arbitrary text tags. A tabular view of a tree is a sortable list of rows, one per node. Sorting its text labels permits rapid navigation of annotated structures of interest in the neuronal arbor. For example, we use ”TODO” labels to tag branch stubs while reconstructing a principal branch, so that these branches are not neglected. Other tags document synapses and other structures of interest (Figure 2b,c of main text).

When the number of reconstructed objects exceeds the hundreds, tracking which objects represent what structure, and with what properties, becomes difficult. TrakEM2 offers an annotation field for each reconstructed object so that information can be stored in direct association with the structure of interest (Figure 2a of main text). All text-containing elements in TrakEM2 are searchable by regular expression, for rapid navigation to the object of interest (Figure 2b of main text).

1.8 Neuronal circuitry reconstruction with skeleton trees and connectors

The reconstruction of a circuit requires on the one hand the reconstruction of its constituent elements, the neurons and glial cells; and on the other hand, the annotation of their connections, the synapses and gap junctions. TrakEM2 offers the data type ”connector” to establish a directional relation between elements. A ”connector” relates a single source structure, such as a presynaptic terminal, with one or more target structures, such as postsynaptic terminals of a polyadic synapse (Figure 2a,h of main text).

The manual reconstruction of neuronal arbors is a very time consuming, and yet it is the current state of the art for serial section EM of complete neuropils (Bock et al., 2011; Briggman et al., 2011). The fastest manual neuronal arbor reconstruction method to date uses trees of parent/child nodes that

represent the skeleton of the arbor (Helmstaedter et al., 2011). TrakEM2 offers two types of skeleton: the "area tree", in which each node is associated to an area, and the "tree line", in which each node has a radius. The former is useful for rapidly sketching the arbor of a neuron and later on filling in the area that represents the sectioned neurite profile. The latter is useful for an approximate representation of the arbor with approximate contours of the branches. Both data types enable trivial correction of split or join errors. A special-purpose graphical interface provides the means for a quick and comprehensive review of reconstructed arbors (Figure 2c,d of main text).

The circuit emerges by relating nodes of multiple "area tree" arbors with "connector" synapses. With TrakEM2, a small *Drosophila* larva interneuron with approximately 120 microns of cable length, 20 presynaptic sites and 20 postsynaptic sites, and existing in over 400 serial sections, is reconstructed by a human operator in less than 2 hours (for an example of a small interneuron, see supplementary movie 1). Complex interneurons with 400 microns of cable and about 150 presynaptic and 150 postsynaptic sites require several days. A disproportionally large effort is required for small terminal dendrites (which are more abundant in larger arbors), and in annotating postsynaptic partners in polyadic synapses.

Each node of a tree carries text tags that label either biological structures of interest such as membrane specializations, or details of the underlying images such as the presence of support film folds and noise. The latter kind of annotations are useful for tuning the initial set of assumptions of an automatic image segmentation algorithm. All tags are searchable for rapid navigation of regions of interest in the reconstructed neuronal arbor (Figure 2b,c of main text).

The "connector" elements that relate two or more objects transform a collection of reconstructed objects into a meaningful graph—the circuit. Connectivity-based edition, analysis and visualization are then possible. For example, all arbors downstream of a specific arbor may be added to the canvas selection and then collectively counted, measured, colored or displayed in 3d.

A common strategy for reconstructing specific circuits is to reconstruct a neuron of interest and then reconstruct every neuron that synapses onto it or receives a synapse from it. TrakEM2 facilitates this task by listing all the synapses of a neuron with their synaptic partners in a sortable table. The task then consists in jumping to each position and reconstructing each neuron, performing a breadth-first reconstruction of the circuit around the neuron of interest. In this manner, the shortest circuit paths between a specific sensory axonal arbor and a specific motorneuron are found with minimal effort.

TrakEM2 exports the reconstructed circuity to the neural network format *NeuroML* (Gleeson et al., 2010). The network is then ready for functional analysis in the neural circuitry simulation software packages Neuron (Carnevale and Hines, 2006), neuroConstruct (Gleeson et al., 2007) or the generic simulator PyNN (Davison et al., 2009); or for morphological and developmental simulations with the

software package CX3D (Zubler and Douglas, 2009).

1.9 Measurements

The hierarchical structure that organizes reconstructions in TrakEM2 enables measurement at the desired level of abstraction, be it a part of a neuron, a single neuron, a group of neurons related by their neuronal lineage, entire compartments or tissues, or more generally any collection of arbitrarily grouped elements (Figure 3 of main text). The measurement of an individual high-order element results in the accumulation of the measurements of all underlying primitive objects that represent the reconstructions (e.g. instances of ”area tree”, ”connector”, ”area list” etc.) in appropriate tables of results, listing counts, lengths, surfaces, and volumes. These tables are exportable as comma-separated values for further processing Fig. S8 f).

The tree data types offer customized measurement methods. For example, to measure the distance of all ”presynaptic site” tags to the root of the tree (Fig. S9 a), to measure the distance of all presynaptic sites (as labeled with ”connectors”) to the root or a marked node, to measure lengths between two chosen tree nodes, or to measure the lengths between all existing specific tag pairs. The latter is useful for example for quantifying the length, diameters or volumes of spine necks when suitably labeled.

The ”dissector” data type enables the estimation of object densities (such as synapses) in the volume with the double disector method (Geinisman et al., 1996) (Fig. S9 c). A grid overlay parcels the 2d image space for sampling or as a reference (Fig. S9 c). The ”polyline” data type, among other purposes, is useful for quantifying lengths of any structure across 3d space (not shown).

TrakEM2 is embedded in the image processing environment provided by ImageJ (Rasband, 2011) and Fiji (`http://fiji.sc`). ImageJ provides a number of region of interest (ROI) tools for measuring and quantifying images. All of these ROI tools are accessible from TrakEM2 for direct calibrated measurements of structures in two dimensions. Measurements in 3d are performed with TrakEM2’s primitive data type ”polyline” (for lengths) and ”area list” (for volumes), or with any other suitable data type.

1.10 Customizing TrakEM2 for special-purpose applications

The open source nature of TrakEM2 enables customizations at all levels. While the most common are scripts and additional graphical interfaces, the source repository may be forked and modified at will for any special purpose (for an example see Bock et al., 2011). Furthermore, any potential problems with

the application itself or requests for new features are addressed within days, powered by the continuous deployment model offered by the host image processing environment Fiji (Schindelin, 2008).

Every operation accessible to the human on the graphical display is also available for programs to execute. Any language available for the JVM is capable of invoking functions in TrakEM2's internal data structures and libraries. We favor the python scripting language and we have elaborated an extensive introduction to hacking TrakEM2 with python in wiki format (`http://fiji.sc/Jython_Scripting`). Common uses include customized means to import image collections, special-purpose coloring of segmentations, object annotation, and batch processing of image tiles or segmentation elements (for an example of a script see Fig. S9 d).

The graphical interface itself is extensible for special purpose applications. For example a new tab may be added to host special purpose fields for entering data and applying operations to selected objects. ImageJ and Java libraries provide any desired graphical widget for further customizations.

References

Arganda-Carreras, I., Sorzano, C. O. S., Marabini, R., et al. (2006). Consistent and elastic registration of histological sections using vector-spline regularization. In *Computer Vision Approaches to Medical Image Analysis*, volume 4241 of *Lecture Notes in Computer Science*, pages 85–95, Berlin / Heidelberg, GER. Springer.

Betzig, E., Patterson, G., Sougrat, R., Lindwasser, O., Olenych, S., Bonifacino, J., Davidson, M., Lippincott-Schwartz, J., and Hess, H. (2006). Imaging intracellular fluorescent proteins at nanometer resolution. *Science*, 313(5793):1642–5.

Bock, D., Lee, W., Kerlin, A., Andermann, M., Hood, G., Wetzel, A., Yurgenson, S., Soucy, E., Kim, H., and Reid, R. (2011). Network anatomy and in vivo physiology of visual cortical neurons. *Nature*, 47(7337):177–82.

Breiman, L. (2001). Random Forests. *Machine Learning*, 45(1):5–32.

Briggman, K. L., Helmstaedter, M., and Denk, W. (2011). Wiring specificity in the direction-selectivity circuit of the retina. *Nature*, 471:183–8.

Cardona, A., Saalfeld, S., Arganda Carreras, I., Pereanu, W., Schindelin, J., and Hartenstein, V. (2010). Identifying neuronal lineages of *Drosophila* by sequence analysis of axon tracts. *J Neurosci*, 30(22):7538–53.

Carnevale, N. and Hines, M. (2006). *The NEURON Book*. Cambridge University Press, Cambridge, UK.

Chklovskii, D., Vitaladevuni, S., and Scheffer, L. (2010). Semi-automated reconstruction of neural circuits using electron microscopy. *Current Opinion in Neurobiology*, 20(5):667–75.

Cortes, C. and Vapnik, V. (1995). Support-vector networks. *Machine Learning*, 20:273–297. 10.1007/BF00994018.

Davison, A. P., Bruderle, D., Eppler, J. M., Kremkow, J., Muller, E., Pecevski, D., Perrinet, L., and Yger, P. (2009). Pynn: a common interface for neuronal network simulators. *Frontiers in Neuroinformatics*, 2(0).

Fiala, J. (2005). Reconstruct: a free editor for serial section microscopy. *J. Microscopy*, 218:52–61.

Fischler, M. A. and Bolles, R. C. (1981). Random sample consensus: a paradigm for model fitting with applications to image analysis and automated cartography. *Communications of the ACM*, 24(6):381–95.

Geinisman, Y., Gundersen, H., van der Zee, E., and West, M. (1996). Unbiased stereological estimation of the total numberof synapses in a brain region. *Journal of Neurocytology*, 25:805–19.

Gleeson, P., Crook, S., Cannon, R. C., Hines, M. L., Billings, G. O., Farinella, M., Morse, T. M., Davison, A. P., Ray, S., Bhalla, U. S., Barnes, S. R., Dimitrova, Y. D., and Silver, R. A. (2010). Neuroml: A language for describing data driven models of neurons and networks with a high degree of biological detail. *PLoS Comput Biol*, 6(6):e1000815.

Gleeson, P., Steuber, V., and Silver, A. R. (2007). neuroConstruct: A Tool for Modeling Networks of Neurons in 3D Space. *Neuron*, 54(2):219–35.

Helmstaedter, M., Briggman, K., and Denk, W. (2011). High-accuracy neurite reconstruction for high-throughput neuroanatomy. *Nature Neuroscience*, 14(8):1081–8.

Jain, V., Seung, H., and Turaga, C. (2010). Machines that learn to segment images: a crucial technology for connectomics. *Current Opinion in Neurobiology*, 20(5):653–66.

Kaynig, V., Fischer, B., Muller, E., and Buhmann, J. (2010a). Fully Automatic Stitching and Distortion Correction of Transmission Electron Microscope Images. *Journal of Structural Biology*, 171(2):163–73.

Kaynig, V., Fischer, B., Muller, E., and Buhmann, J. (2010b). Neuron Geometry Extraction by Perceptual Grouping in ssTEM Images. *CVPR*.

Kaynig, V., Fuchs, T., and Buhmann, J. (2010c). Geometrical consistent 3D tracing of neuronal processes in ssTEM data. *Medical Image Computing and Computer-Assisted Intervention*, 6362:209–216.

Klar, T., Jakobs, S., Dyba, M., Egner, A., and Hell, S. (2000). Fluorescence microscopy with diffraction resolution limit broken by stimulated emission. *Proc Natl Acad Sci USA*, 97:8206–8210.

LeCun, Y., Boser, B., Denker, J., Henderson, D., Howard, R., and Hubbard, W. (1989). Backpropagation applied to handwritten zip code recognition. *Neural Computation*, 1:541–51.

Longair, M., Baker, D., and Armstrong, J. (2011). Simple Neurite Tracer: Open Source software for reconstruction, visualization and analysis of neuronal processes. *Bioinformatics*.

Lorensen, W. E. and Cline, H. E. (1987). Marching Cubes: A high resolution 3D surface construction algorithm. *ACM SIGGRAPH Computer Graphics*, 21(4):163–169.

Lowe, D. G. (2004). Distinctive image features from scale-invariant keypoints. *International Journal of Computer Vision*, 60(2):91–110.

Mastronarde, D. (2005). Automated electron microscope tomography using robust prediction of specimen movements. *J Struct Biol*, 152:36–51.

Preibisch, S., Saalfeld, S., and Tomancak, P. (2009). Globally optimal stitching of tiled 3D microscopic image acquisitions. *Bioinformatics*, 25(11):1463–5.

Rasband, W. (1997-2011). ImageJ. http://rsb.info.nih.gov/ij/.

Saalfeld, S., Cardona, A., Hartenstein, V., , and Tomancak, P. (2010). As-rigid-as-possible mosaicking and serial section registration of large ssTEM datasets. *Bioinformatics*, 26(12):i57–i63.

Schindelin, J. (2008). Fiji is just ImageJ – batteries included. In *Proceedings of the 2nd ImageJ User and Developer Conference*, Luxembourg.

Schmid, B., Schindelin, J., Cardona, A., Longair, M., and Heinsenberg, M. (2010). A high-level 3 D visualization API for Java and ImageJ. *BMC Bioinformatics*, 11:274.

Sethian, J. (1996). A fast marching level set method for monotonically advancing fronts. *Proc Natl Acad Sci USA*, 93:1591–1595.

Sommer, C., Straehle, C., Köthe, U., and Hamprecht, F. A. (2011). "ilastik: Interactive learning and segmentation toolkit". In *8th IEEE International Symposium on Biomedical Imaging (ISBI 2011), in press*.

Suloway, C., Pulokas, J., Fellmann, D., Cheng, A., Guerra, F., Quispe, Stagg, S., Potter, C., and Carragher, B. (2005). Automated molecular microscopy: the new Leginon system. *Journal of Structural Biology*, 151:41–60.

Turaga, S., Murray, J., Jain, V., Roth, F., Helmstaedter, M., Briggman, K., Denk, W., and Seung, H. (2010). Convolutional networks can learn to generate affinity graphs for image segmentation. *Neural Computation*, 22(2):511–38.

Williams, L. (1983). Pyramidal parametrics. In *SIGGRAPH '83: Proceedings of the 10th annual conference on Computer graphics and interactive techniques*, pages 1–11, New York, NY, USA. ACM.

Zubler, F. and Douglas, R. (2009). A framework for modeling the growth and development of neurons and networks. *Frontiers in Computational Neuroscience*, 3(0).

Zuiderveld, K. (1994). *Contrast limited adaptive histogram equalization. Graphics gems IV*. Academic Press Professional, Inc., San Diego, CA, USA.
